# Rituals as Nature-Based Governance of reciprocity between people and nature

**DOI:** 10.12688/openreseurope.17206.1

**Published:** 2024-04-12

**Authors:** Carsten Herrmann-Pillath

**Affiliations:** 1 Max Weber Centre for Advanced Cultural and Social Studies, University of Erfurt, Erfurt, Thuringia, Germany

**Keywords:** ritual as nature-based governance; interchange between people and nature; possession as more-than-human ritual; ritual governance of nature-based solutions

## Abstract

The conventional approach to environmental governance, based on institutions, regulations, and interventions, has utterly failed to stop the current ecological catastrophe. I suggest a radical alternative: Ritual as the core mode of ‘nature-based governance’ (NBG) that enacts deep and comprehensive reciprocity between people and nature. NBG grounds governance mechanisms in embodied more-than-human practices with normative force. I combine a wide range of theoretical resources in social sciences, economics, and philosophy to suggest a general concept of ritual that is inspired by but generalizes over Indigenous thought and is informed by East Asian ideas about ritual as the pivot of social order. However, the radical basis for my argument recognizes ritual as a kind of action that humans and non-humans share as living beings. Therefore, rituals can be activated in workable governance mechanisms to create and sustain communities of multi-species cohabitation. I present a theoretical case study on property as ritual; this relates human property of land with non-human territoriality, acknowledging possession and its ritual performance as a behaviour shared in humans and non-human species. Consequently, rooted in ritual, a more-than-human notion of property emerges that radically differs from modern ideas of the institution of property but converges with Indigenous relational concepts. Equipped with these theoretical insights, I suggest practical applications in the context of NBG of Nature-based solutions. These are: reconceptualizing eco-compensation as a reciprocal ritual of gift-giving, the commoning of ecosystem services of animal populations in wildfire protection, and the formation of urban multi-species communities in urban gardening.

## 1. Introduction: Ritual as a mode of governance

This paper advances a radical and daring claim: Governance of relationships between nature and people should put rituals at its core and laws, regulations, etc., on a secondary position. I refer to this approach to governance as ‘nature-based governance’ (NBG), with rituals being one, though pivotal manifestation. The terminological innovation of NBG was suggested by members of the COEVOLVERS project (Beskydy team, led by Tatiana Kluvankova) when preparing a workshop on Earth System Governance about governance on nature-based solutions ( https://www.earthsystemgovernance.org/news/2024-beskydy-workshop-call-for-papers/). I expand its meaning substantially, on par with other approaches to governance labeled by specific epithets, such as ‘adaptive’ or ‘global’ ( Bennett & Satterfield, 2018).

My use of the term ‘nature’ is philosophically reflected: It aims at overcoming the human/nature divide that is still haunting much thinking about ecology and society in practice, such as in the notion of ‘nature-based solutions’ which adopt an instrumental approach to nature as distinct from the human domain ( Seddon *et al.*, 2021). ‘Nature-based’ in this paper approaches humans as part of nature, recognizing that in the transition to the Anthropocene, the human domain has expanded to effectively include the biosphere in its range, obliterating the division between nature and the human, especially including human-built artifacts ( Vogel, 2015). I do not separate the human domain in terms of ‘non-natural’ institutions in governance, apparently defining its autonomy from nature. To the contrary, I claim that ritual is an institutional form that transcends the human/non-human divide and, therefore, can govern human behaviour as critical constituent of ecosystem performance and evolution.

The background of this theoretical innovation is the growing awareness that the relationship between nature and people must be reciprocal ( Ojeda *et al.*, 2022). This idea is often associated with Indigenous thought but has been stated as a general requirement in designing Nature-based solutions as distinct from ecosystem services ( Maller, 2021). The latter are often conceived as contributing to goals defined by the needs of human societies, to which the service is rendered ( Gomez-Baggethun, 2018). Protecting and nurturing the ecosystem is a mere functional requirement of sustaining the capacities for generating these services (‘natural capital’). In the notion of nature-based solutions (NBS), the idea is that there is a reciprocal relationship between people benefitting from nature and nature itself, mostly conceived in abstract terms as sustaining ‘biodiversity’ ( IUCN, 2020). However, this is merely a standard for assessing the performance of an NBS, and the notion of reciprocity is devoid of any substantial meaning in terms of the type of interaction. This is where the notion of ritual comes into play since reciprocity is a term thoroughly explored in anthropology, where it is often seen as being ritually performed, such as in rituals of gift-giving. I posit that NBG conceptualizes reciprocity rigorously, and not just metaphorically, as a flow of gifts between nature and people.

My claim is based on a wide range of philosophical, anthropological, and sociological contributions. It is motivated by the empirical diagnosis that the conventional approach to environmental protection and policies has failed to meet our times' challenges. This is especially true for biodiversity. Something is fundamentally wrong in our relationship ‘with nature’. The reason is that speciesism deeply permeates our modern worldview and most scientific thinking in viewing the human species as being distinct in some essential respects from all other species ( Singer & Harari, 2023). This combines with anthropocentrism in approaching human action and policies worldwide, such as maintaining this position when analysing ecosystem services ( Dasgupta, 2021). The separateness of humans is grounded in ideas about the uniqueness of the human mind and culture, which not only downgrade the position of other species but also reinforce a Cartesian denigration and devaluation of the human body in ideas about mind and rationality. After all, it is ‘being a body’ (and not ‘having’) which is a fundamental feature that is shared with all other species, and the paradigm of dominating other species goes hand in hand with the idea of dominating the human body by rational thought and rational institutions.

Ritual is the foundational analytical category that breaks through speciesist constraints in our thinking about a more-than-human community. Ordering interaction between living beings by ritual is a biological universal ( Stephenson, 2015). Hence, ritual is the obvious candidate if we search for a most general category that could apply to governing people’s relationship with nature. Whereas we cannot conceive of law as being shared between human and non-human species, ritual is one category that applies to both. There are others, such as play; but I leave this for other work. However, ritual includes many of those, for example, since play often includes ritualized performances. The same is true for art since ritual is also an aesthetic practice ( Prum, 2017).

Let us preliminarily define ‘ritual’ as a specific type of action ( Bell, 1997; Collins, 2005). Ritual is a regularized behavioural pattern that is recurrent through time, however, with no fixed schedule, often triggered by certain events which are themselves determined by the ritual: I refer to this as ritual affordances (this includes calendar rites, biological rhythms, types of encounters, or life-cycle events, among others). There are individual rituals (such as those related to cleaning and hygiene) and collective ones, which include a wide range of types of interaction. In the case of humans, ritual affordances often include artifacts specifically designed for that purpose. A key feature of ritual is that the regularity is deeply embodied; there is only marginal engagement of rational reflection and other mechanisms of choice. Ritual is pure action. However, this does not mean there is no reflective element that would apply to purely automatic behavior as a distinct type. An important aspect of human ritual is the sincere deployment of the ritual practice, and less the cognitive states accompanying the actions ( Seligman *et al.*, 2008). In other words, the specific reasons why ritual is deployed are not essential, but proper and exact enactment of ritual (orthopraxy as opposed to orthodoxy). This critical role of proper doing the ritual is the key to establishing ritual as a concept that applies uniformly across humans and non-humans.

Ritual is almost synonymous with ordering behaviour and is, therefore, an essential form of governance, which is, however, neglected in established institutional approaches to governance, where it is mainly seen as cultural means to create legitimacy to institutions, such as conveying an aura of sanctity to a judge. This view is the consequence of the distinct cultural history of ritual in Western modernity ( Asad, 1993). The neglect of ritual in contemporary social thought reflects what Charles Taylor (2007) has called the ‘excarnation’ of Western societies in the aftermath of the Reformation, that is, the growing compartmentalization between embodied and disembodied spheres of life, with the disembodied spheres being the domains of rationality (bureaucracy, market, science, etc.). This view is mirrored in Max Weber’s influential approach to modernization as rationalization. Tellingly, Weber struggled with understanding the role of ritual in China, which he qualified as ‘enchantment’ and the antipode of Western rationality as ’disenchantment’ ( Yang, 2020). For him, Confucian ritualism was a major reason China did not transform into capitalism, even though it had appeared as the most advanced stage of civilization to Enlightenment thinkers such as Wolff, Quesnay and Voltaire.

I mention this point because, in the context of ecology, ritual is mostly associated with so-called ‘indigenous’ thought, a category that would exclude many societies in which ritual has been a dominant mode of ordering society, such as China ( Herrmann-Pillath, 2016a). In ecological philosophy, the key idea is that Indigenous people maintain relational values and practices with nature, embedded into cultural imaginaries such as myths ( Alexander, 2013). Although this is important and inspiring, the question remains how to close the gap between mainstream thinking in the sciences and the humanities? Further, there is the exclusive juxtaposition between the Indigenous world and the so-called ‘Western’ societies, which ignores the cultural diversity across the world independent from the level of economic development. One of the inspirations of this paper is the comparison between East Asia and Western cultures in the critical dimension of ritual. Traditionally, before undergoing sometimes violent transformations of modernization, social order in East Asia was grounded in ritual. However, strong aspects of ritual persist and often are defining features of cultural identity in difference to the so-called ‘West,’ as in the case of Japan ( Nelson, 1992). So, although Indigenous thought and practice is an important inspiration for conceiving ritual as the key of NBG, the case for it has a much wider frame of reference, in which the Western marginalization of ritual appears as the exception, not the rule. As in the Japanese example, Shinto ritual has deep potential for creating NBG of NBS ( Ishizawa, 2018).

The next section presents a brief exploration of the theoretical foundations of ritual as NBG. Next, we look at property as a theoretical case study to nail down the difference between institutions in the common view and ritual. Section 4 discusses applications in the NBS context. Section 5 provides conclusions.

## 2. The intellectual sources of Nature-Based Governance

In environmental policies, governance refers to all institutional and administrative means that enforce policies and laws intended to resolve environmental dysfunctions in the economy and society ( Young, 2013). The most general meaning of governance is enforcing and monitoring the ‘rules of the game’. This definition leaves open the question of who enforces and monitors. Diverging answers is one reason why different disciplines and fields of application give different meanings to the terms on a more specific level. One important strand is to approach governance as a form of government that transcends conventional hierarchical-bureaucratic modes and activates societal forces and groups to achieve public goals ( Treib *et al.*, 2007). Another strand conceives governance in more articulate opposition to government and as a form of social self-organization, especially in community governance. Further, notions of governance may emphasize different aspects, in particular, efficiency versus equity ( Bennett & Satterfield, 2018): The former emphasizes performance, effectiveness, and resilience, and the latter emphasizes justice, inclusion, and democracy.

The literature on governance often approaches the term as if it implicitly has an independent theoretical status, in the sense of a ‘theory of governance,’ which differentiates into various divisions such as domains or modes of governance. However, such approaches tend to leave behavioural foundations out of sight and approach governance only in terms of structures, institutions, and mechanisms, such as bureaucracy, networks, or markets ( Pahl-Wostl, 2019). This incompleteness may explain why economics often lurks behind theories of governance since economics offers an integrated view of behaviour and institutions.

Economic paradigms have strongly influenced the established theory of governance in that institutions are often treated as analytically independent from actors who respond rationally to institutionalized incentives ( Williamson, 1985). These can be modulated by cognitive factors such as worldviews and ideologies ( North, 2005). Economic theories count in the context of ecology because most ecological challenges result from economic action and, therefore, require economic governance mechanisms that are at least complementary to others. However, this also creates deep tensions when combined with other approaches to governance that emphasize community aspects, inclusion, or care. What needs to be improved is an integrative theory of governance since otherwise, practices of governance fail because the different approaches rest on fundamentally conflicting anthropological and behavioral assumptions.

Institutional economics distinguishes between formal and informal institutions, which is also essential in governance. Formal institutions include environmental law and the administrative measures of enforcing and monitoring it. This need be not only governmental institutions but also measures such as voluntary agreements on labeling eco-friendly products, which are enforced and monitored by private-sector agencies. Informal institutions include various forms of governance in social groups, such as observing rules of conduct of group members, for example, neighbourhoods or peer groups. A question is how far internalized norms are included in these notions. For example, the socialization of children may proceed in a formalized institutional setting, but eventually, the norms become internalized values that later are no longer formally enforced.

This observation alerts us of the significant difficulty that results from neglecting the behavioural foundations of governance. Economics assumes rational actors, which implies parsimonious incentive-based approaches to understanding and designing governance. However, if people are value-driven, emotionally inspired, or altruistic, the implications for governance would differ widely. To add complications, we know that governance mechanisms are performative in the sense that a particular type of governance may also generate a particular type of actor, contrary to the universalist claims of rational choice paradigms ( Herrmann-Pillath, 2016b).

Where do we locate ritual in this theoretical canvas? Two observations need attention. The first is that ritual does not easily fit into the categorizations of institutions because all aspects count in a complex way. First, many rituals are formalized, often in great detail, and some bodies monitor and enforce proper rituals, such as in churches as a form of religious community. However, this is not a necessary feature of ritual, as second, ritual is often observed in communities without external enforcement, but violating ritual expectations may trigger strong social sanctions, including ostracism. Third, ritual is internalized chiefly because proper adherence to ritual is an essential component of individual identity as a group member, which also grounds spontaneous group sanctions of improper behaviour. These three points show that ritual is a phenomenon sui generis, crosscutting the analytical distinctions in standard institutional approaches to governance. In ritual, the analytical distinction between the actors and the institution becomes obsolete.

The second observation is that institutions are widely conceived as distinctly human. We would not count behavioural regularities in baboon troops as ‘institutions’ and approach hierarchies in those groups as ‘governance structures’. There are various reasons. One is that human institutions appear to be culturally contingent, hence variable independent from genetic factors, whereas non-human regularities are seen as genetically determined. Another reason is that institutions are mediated mainly by language, which is conceived as distinctly human ( Searle, 2011). In this sense, governance could not be ‘nature-based.’ This view is a problem regarding environmental issues since established uses of ‘environmental governance’ tend to include the ecosystem when assessing governance performance but exclude the non-human actors from the reference domain of ‘governance’.

If we combine the two observations, we get the inspiration that ritual may be an alternative form of governance not affected by the claims on human distinctiveness related to institutions. Whether we do so depends on how we further dissect the notion of ritual and which weight we give to the second and third points above: the role of social sanctions and internalization in ritual governance. Following the introduction, this relates to the more fundamental question of to which extent embodiment grounds governance, more-than-human. This approach is ‘nature-based governance’:

*Nature-based governance grounds governance mechanisms in embodied more-than-human practices with normative force*

We disentangle this highly compressed preliminary definition into various components related to specific social science literature strands. These strands are partly independent and disconnected from each other. Viewing them together provides an innovative theoretical foundation for NBG (building on Basso & Herrmann-Pillath, 2024).

- The first strand is classical French critical thinking. We emphasize Foucault and Bourdieu since both contributed to the study of embodiment in governance mechanisms. Both theorists have emphasized the role of internalizing institutions. As for Foucault, the key concept is 'governmentality', which refers to how government can promote values and behavioural norms such that the governed obey regulatory powers without reflection or even awareness ( Foucault *et al.*, 2004). One example is the internalization of hygienic standards in the 19 ^th^ century. This matches with Bourdieu's notion of 'habitus', which is broader as it does not only refer to governmental power but more generally to social status and hierarchy ( Bourdieu, 2019). Habitus is a strong force in regulating behaviour because it forms a person's identity, especially as a member of a particular group in society. Habitus internalizes governance via the unequal distribution of cultural capital in society, among other factors.
- Embodiment is also an essential aspect of the second strand, the modern theories of behavioural economics ( Thaler, 2016). 'Nudging' is a concept that shares strong family resemblances with governmentality: The idea is that a regulatory power shapes the environment of individual choice such that, independent from deliberate choice, the desired behaviour is generated. Behavioural economics differs from standard economics in eschewing the model of the unified rational actor, which complicates the view on mechanisms that link incentives and behaviour. One influential approach is the two-systems view, which directly ties up with the Cartesian mind-body duality in positing two systems, rational and affective ( Kahneman, 2012). Nudging addresses the latter to achieve societal goals that harmonize with the rational system.
- This approach ties up with a third strand, theories of distributed agency. Whereas the classical theories of institutional governance assume an autonomous individual decision maker, distributed agency approaches agency as emerging in assemblages of individuals and material entities ( Malafouris, 2013). Although the observed behaviour is empirically manifest in individuals, the underlying agency is materially distributed. In this case, following Basso and Herrmann-Pillath, we distinguish between inward and outward embodiment: The former stands in line with the Foucault and Bourdieu views, and the latter emphasizes the role of assemblages in generating behaviour. A substantial and varied literature develops this aspect, such as actor-network theory ( Latour, 2005).
- Another, fourth strand engages biology and behavioural sciences. From many angles, the culture/nature dichotomy that informs standard theories of institutions has become obsolete, including specific approaches such as theories of coevolution ( Jablonka *et al*., 2014) or evolutionary psychology ( Reader & Workman, 2024). These have internal tensions, and not all of them are equally relevant. The critical point here is that they endorse the case for a biological grounding of human culture while not denying species-specific characteristics of humans.
- The fifth strand works in the opposite direction. This is the increasing recognition of non-human forms of sociality and cognition that are functionally equivalent yet radically different from human forms ( Godfrey-Smith, 2020). One crucial aspect is the universality of forms of sentience, which provide the ground for weakening speciesist claims even in the case of institutions, such as the law ( Deckha, 2021).
- The sixth strand is the vast literature on embodied cognition which undermines claims of human distinctness as relating to an ‘amodal’ domain of human cognition epitomized in rationality and logical thought claimed to be independent from material substrate ( Johnson, 2017). Theories of embodiment posit that even abstract concepts such as mathematics are grounded in sensorimotor circuits and re-enactments of action arranged in complex layers and networks of reflexive processing.

This list could be continued (for example, there is the tradition of pragmatism of Peirce and Dewey). However, for my current argument, the six entries already present overwhelming evidence that the standard view on institutions needs radical rethinking.

Where can we locate ritual in this vast universe of discourses? One line of thinking relates ritual to biological roots as a deeply embodied form of human behaviour that enables the distinctly human form of ultrasociality ( Rossano, 2016). Ritualization is a primary form of behavioural coordination in animals, that is genetically endowed, but in terms of activation shaped by epigenetic mechanisms, learning by imprinting and therefore allowing for contextualization beyond genetical fixation. A standard example is the ritualization of birdsong, which is genetically endowed but learned in a specific population context to show great intraspecies variety across local bird habitats. Birdsong is a medium that literally orchestrates intra- and interspecies cohabitation in a specific territory ( Despret, 2019). In this sense, ritual is a coevolutionary phenomenon that leaves much leeway for forms of behaviour which transcend genetic determination and converge to human forms of culture, such as in aesthetic practices ( Prum, 2017).

Ritual ties up with critical notions such as embodiment, distributed agency, or habitus in playing an essential role in forming human communities and the identities of their members, grounded in emotional attachments and shared practices of expressing them. The influential Durkheimian view sees ritual as the cement of community, especially in religion ( Durkheim, 2012). Goffman represents another line of thinking that explores the role of rituals in everyday life and even apparently minor encounters ( Goffman, 1982). One recent approach integrating these views is Randall Collins’ theory, in which rituals are fundamental in ordering social interactions by mobilizing and sharing emotional states among actors ( Collins, 2005). Like with a Necker cube, Collins switches the view on institutions such as the market from the established emphasis on rational design and choice to the view on ritual. His approach implies that we can envisage a frameshift from institutions to rituals across all domains of human sociality. Once this step is done, ritual can also expand the theory of governance to the more-than-human.

All these theoretical contributions cannot be elaborated here in more detail. Since the proof of the pudding is in the eating, we continue with a brief theoretical case study.

## 3. A theoretical case-in-point: Ritual, territory and property

In this section, we consider an essential case for fixing the fundamental conceptual difference between the institutional and the ritual forms of governance. Property is one of the critical institutions in the economy, and designing property rights is seen as an essential regulatory tool in environmental policies, such as in cap-and-trade regimes or in managing common pool resources. In the mainstream approach, property rights are an object of social engineering. Recently, various authors have suggested that property rights should and could also be assigned to animals or ecosystems ( Bradshaw, 2020; Hadley, 2015), reflecting the trend towards recognizing the rights of nature in general ( Chapron *et al.*, 2019). We do not discuss such proposals' merits and possible drawbacks here and only concentrate on adequately conceptualizing property.

The protagonists of animal property point out that this is rooted in biological universals of claiming and controlling resources, most salient in territoriality which bears many resemblances with human claims on land. In the history of land ownership, there was a transition towards regimes in which land is conceived as an abstract parcel on a map that defines the boundaries and identified proprietors: land registries ( Vanuxem, 2022). Before the advent of modern capitalism, such registries were almost exclusively for tax administration. In contrast, the modern civil laws of the 19th century focused on easing land market transactions and using land as collateral in financial transactions. These institutional innovations often deliberately targeted local property institutions such as the commons, which were community-based and rooted in traditional norms of land use ( de Moor, 2018). Since these community norms were also enacted in various customary practices, we can conceive them as forms of ritual governance.

Regarding non-human property, ritual becomes a universal framework for approaching the relationship between resources and claimants if we distinguish neatly between possession and property ( Emerich, 2017) and between the institution of property and the notion of 'property right' as a term in legal and economic theory ( Hodgson, 2015). Possession differs from the property institution because it is embodied and enacted via factually using an object, whereas property is a formalized legal claim independent from use, in principle. In the legal construct of adverse possession, the formal proprietor may lose her right if another claimant uses the object over a certain period and does not complain or express any interest in using the object ( Serkin, 2016). Yet, this criterion leaves two alternative approaches to identifying possession, subject to an important legal debate in 19th-century jurisprudence: Is it sufficient to record a continuing use of an object to assign possession to the user, or must the user express a claim to possess? The latter would be pre-legal in communicating the 'will' to possess.

This debate is highly significant for our topic since the first interpretation can be easily applied to non-humans since all members of an ecosystem use a resource, such as the land where this system is located. The second interpretation points towards the role of non-human signals in expressing possession, referring to all kinds of ritualization of territoriality. Hence, it is not only straightforward to recognize the possession of non-humans but also to understand ritual as the universal form of claiming possession among both humans and non-humans. There is a wider range of performative behaviours, both linguistically and non-linguistically, how possession is recognized and stabilized among human actors without invoking the law ( Rose, 2019).

The role of ritual is certainly neglected in modern legal theory. However, firstly, rituals played a pivotal role in legal history, such as Roman law, which provided many templates for modern law. Secondly, we must distinguish between property as defined by law and 'lived property'. The law becomes activated in case of conflicts that other means cannot resolve. In ordinary circumstances, the property is enacted in many ritualistic behaviours, that is, communicating property without invoking legal language, such as, for example, rules governing trespassing, and often societal practices are recognized legally only ex-post, such as rights of hunting and foraging ( Cooper, 1998). In many societies, actual land ownership arrangements differ from legal prescriptions and reflect the strength of customary obligations that are ritually enacted ( Ellickson, 1994).

In this context, the debate about the legal status of Indigenous claims on land is highly significant. Indigenous lawyers strongly emphasize the radical difference between common law notions of property (this discussion mainly unfolds in former British colonies) and the indigenous relationship with the land ( McNeil, 2017). The latter emphasizes the shared identity of people and land, even to the degree of seeing landscapes as embodiments of hybrid beings, thus blurring the demarcation between the living and the non-living ( Povinelli, 2016). One of the critical distinctions is the inalienability of the land as a sacred homeland, which straightforwardly can be interpreted as a difference between ritual and institutional governance of land and defines a relational understanding of possession ( Keenan, 2015). These Indigenous views also recognize the shared ownership of resources by humans and non-humans: This is a paradigmatic case of NBG by ritual—the relationship with land grounds reciprocity between people and nature.

Summing up, the case of property shows how we can orchestrate the Necker cube frameshift from institutions to rituals in recognizing the embodied nature of property and the essential role of possession as a concept that easily extends to the more-than-human. In the NBG of property, ritual encompasses all forms of ‘doing property’ different from abstract disembodied legal institutions. This does not mean to eschew the latter, as it is also salient when recognizing tribal law as an independent source of jurisprudence at regular courts. However, the formal legal institutions become grounded in and bounded by ritually embodied NBG.

## 4. Practical significance: Ritual in Nature-Based Solutions

Suppose we conceptualize the relationship between people and nature in terms of reciprocity. In that case, the ritual approach suggests the most general notion of gift exchange: In the context of NBS, the ecosystem service activated in the NBS would be conceived as a gift given and not just a service rendered, which creates the obligation to give a gift back in return. Gift-giving differs fundamentally from other types of exchange, mainly market exchange, as the gift-giving enacts an enduring relationship between the two sides. That means implementing an NBS amounts to ritually creating an ecosystem community; hence, it is one of the essential forms of commoning ( Leitheiser *et al.*, 2022). To understand this ritual nature of reciprocity in NBS, we look at eco-compensation first, which is not an NBS per se but often plays a role in the regulatory design of NBS.

### 4.1. Eco-compensation

Eco-compensation is currently a popular regulatory approach to avoiding or minimizing harm done to the environment by human economic activities ( Gastineau *et al.*, 2021; Wang *et al.*, 2022). Formally, this is a case of reciprocity: Humans claim a part of nature and must give something back. As a formal principle, this includes a wide range of actions, which do not necessarily imply that a functional equivalent of the original state of nature is restored, depending on how reciprocity is formally defined. There are scenarios where the link between harm and compensation is remote, such as clearing a forest in Europe and buying offsets for forestation in the Global South. Most thinking on eco-compensation is economical in that some notion of equal value is invoked, though not necessarily in monetary terms: a salient example is carbon offsets, where the quantity of carbon is the standard of value.

The perspective on ritual changes the approach to eco-compensation radically and directly reframes it in terms of NBG of NBS. Let us discuss this by taking urban land as an example, thus continuing with our discussion of property ( Hiedanpää *et al.*, 2023). The land is a resource that is part and parcel of the local ecosystem, and even if there is no legal recognition, all members of that ecosystem enjoy possession of the land. If urban green space is redeveloped, for example, into a parking lot, humans harm all other members at that location by confiscating their possessions. The redevelopment could be treated along the lines of legal prescriptions on public requisitioning of land, now including the interests of non-human stakeholders ( Hernandez-Santin *et al.*, 2023). However, for example, financial compensation is only applicable to humans.

**Figure 1.**
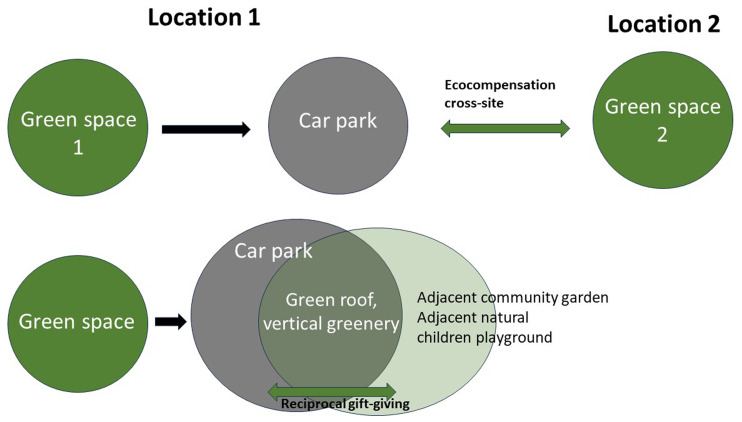
Eco-compensation: Institutional versus ritual governance.

In the ritual approach of NBG, we conceptualize the human appropriation of green space as the reciprocal relationship of gift exchange. The ritual notion of gift exchange is that gifts never lose their attachment to the original gift-giver, in the sense that they remain imbued by her identity and that the gift serves the purpose of expressing and cementing an enduring relationship between the gift-giver and -taker ( Hénaff, 2002). The critical difference to the regulatory notion of eco-compensation is that claiming green space for the human-built environment does not give something back to the ecosystem of equal value. That compensation even severs the link between humans and the ecosystem. In building a parking lot, the identity of the local ecosystem permanently changes. Hence, there is no way to compensate for this harm, for example, by creating a green space in another part of the city (see Figure 1, top). A genuine reciprocal exchange must be enacted in the same ecosystem, matching with ritual obligations of mutual gifts flow. Interestingly, this argument can be supported by considering the role of humans who are also suffering from the intervention and cannot enjoy benefits from eco-compensation, for example, young children and older adults who previously enjoyed the green space and cannot get access to the compensatory area at another place in the city. For them, destroying the green space means a permanent change in their way of life.

The ritual approach requires enacting local reciprocity ( Figure 1, bottom). In the example of the parking lot, one solution is to employ a biophilic architectural design of the parking lot ( Evans & Hardman, 2023). One option is to build a multi-story car park with vertical greenery, green roofs, and adjacent greenery that forms an alternative integrated green space that would match the needs of ecosystem members in the original green space. In general, this example points to the possibility of implementing a reciprocal exchange in which harm done to the local ecosystem is reciprocally balanced by designing the 'grey' artifacts to offer support and nurturing for nature. In a more detailed design, this would manifest in various ritual forms, such as neighbourhood stewardship for plants and animals living in the microhabitat or seasonal neighbourhood festivals. This might especially engage the vulnerable groups with no alternatives in the conventional eco-compensation approach.

### 4.2. Wildfire protection

**Figure 2.**
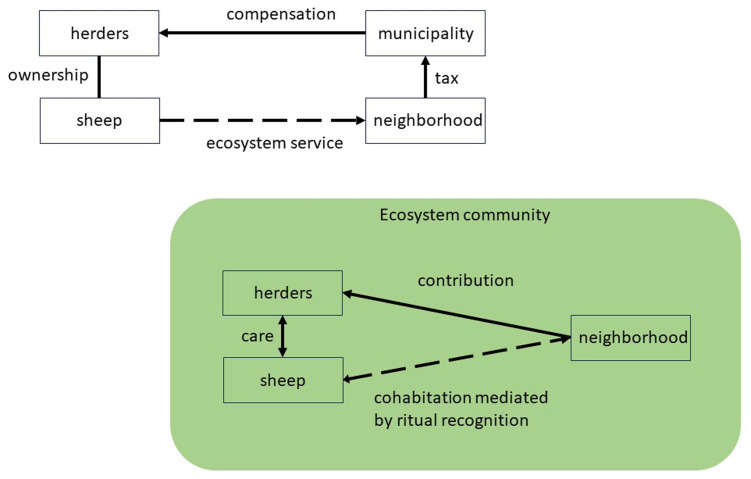
Wildfire protection as ecosystem service versus ritual governance of common good.

The second example of the ritual approach to NBS is using animals in wildfire protection, such as sheep grazing in areas bordering forests and human settlements ( Ribeiro *et al.*, 2023). A standard approach views this as a problem of compensating herders for rendering the service to keep, monitor, and protect the sheep. That means the regulatory arrangement is only among humans and does not include the non-humans as stakeholders or parties. For example, the neighborhood may pay a fee to the municipality that arranges the service managed by herders ( Figure 2, top). There is no relationship between people in the neighbourhood and the sheep who generate the ecosystem service of grazing. On the contrary, sheep may often be counted as a nuisance.

The ritual perspective suggests the radical shift to regard the animals as members of the arrangement with full rights. In principle, administrators may recognize the sheep in institutional terms by assigning legal status to them and representing them by human stewards. However, the relationship quality between people in the neighbourhood and animals would remain the same. Ritual is transformative as it would enact a process of commoning, building, and sustaining a community of sheep and humans, especially those in the urban neighbourhood who ultimately benefit from the ecosystem service. From the economic standpoint, all community members benefit from a common good: wildfire protection. Commoning enacts this mutual sharing of benefits as another form of gift exchange, which includes transforming the fee in the regulatory approach into a contribution paid directly to the herders for their engagement ( Figure 2, bottom). However, ritual adds many aspects of immediate recognition of animals as members of the local ecosystem community, which includes the forest, the border area, and the urban neighbourhood.

For example, rituals include all forms of story-telling that express a relationship between humans and animals or all variants of direct mutual engagement of humans and animals. An example is practicing the welcoming of newly born members of the community: Humans may celebrate the birth of sheep and recognize their individuality as members of the community, for example, by giving them names. Settings of ritual practice can be preschool and primary school institutions, where parents are typically involved in activities, thus also engaging adults. Rituals engaging human children and sheep create long-term memories and mutual bondings that eventually sustain the commoning of the NBS.

### 4.3. Urban gardening

**Figure 3.**
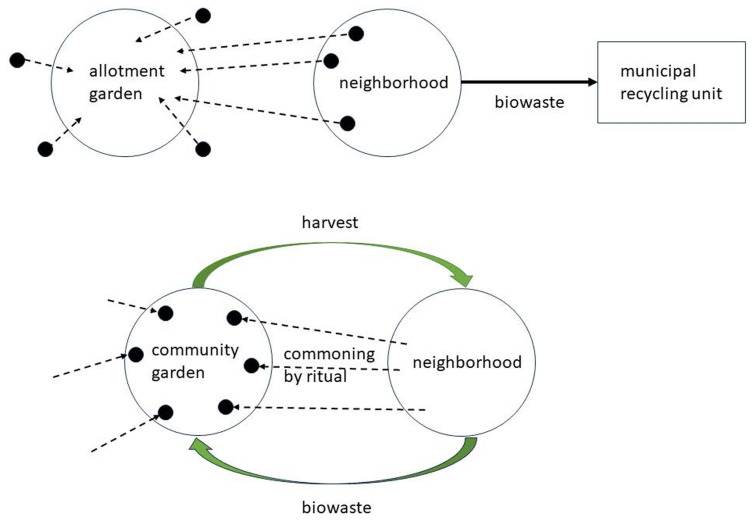
Two forms of urban gardening.

The third example is urban gardens. Urban gardening has a rich and diverse tradition in many forms, such as parks, community gardens, or household lots. Gardening can be seen as NBS in various functional contexts, such as stabilizing urban microclimate or contributing to public health ( Kingsley *et al.*, 2021). Let us consider one variant: a community garden maintained by a local group of human hobbyists. Community gardens can take different institutional shapes, such as allotment gardens or collectively managed schemes. Community gardens claim valuable urban space and are always potentially threatened by urban development. As we saw, an alleged solution is to implement an eco-compensation scheme. Often, this is endorsed even by the neighbourhood since the garden users may be a minority or even partly outsiders ( Figure 3, top), which is especially true for allotment gardens, where the beneficiaries of the institution are exclusively the individual holders of lots. In contrast, the ritual approach to commoning creates a setting of mutual flows of reciprocal gifts between the various parties of the local ecosystem. It emphasizes the collective management of the garden.

One example of the potential for ritualization is the essential role of composting, which needs the input of organic wastes ( Puig de la Bellacasa, 2017). A principle of modern waste management is to make waste invisible to urbanites; hence, even when it comes to organic waste, it is collected in bins and transported to industrial composting sites ( Figure 3, top). The ritual approach suggests creating flows of gifts between the garden and the neighbourhood, where the latter sends the gift of organic waste to the garden, and the garden returns gifts, such as vegetables and fruits, for example, at certain communal festive events ( Figure 3, bottom). The flow of gifts connects the community garden to the neighbourhood, which is independent of the actual engagement of all residents in the garden. Gardens can be rooted in the community in many other ways, such as beekeeping. Even for the bees, a similar mechanism of creating ritual relationships is feasible as for the previous case of the sheep, that is, individualizing bees by tagging and making them visible to neighbours as individual visitors to their balconies ( Chittka, 2024) https://www.pollinatinglondontogether.com/.

Gardening is a crucial example, if not a paradigm for nature-based governance: A human gardener must pay attention to the natural conditions and requirements of the garden and hence is 'governed' by its rules ( Schwarz, 2023). Without giving back and caring, the garden cannot flourish. Composting is a vital activity that is made invisible in what has been aptly referred to as 'sensory governance' in urban infrastructure ( Schulte-Römer, 2023), as in this case, rendering waste invisible and separated from the living eco-community, staying in the long tradition of urban governmentality for public order and sanitary improvements. Making organic fertilizing possible via ritual flows of biowaste gifts changes this pattern of sensory governance in raising awareness among all residents about the connectivities in the local ecosystem.

## 5. Discussion and conclusion

The concept of NBG rests on two pillars. The first is the peculiar anthropology of embodiment, that is, a distinct view of human nature that eschews disembodied approaches to governance that emphasize rational and abstract design and generalized incentives. This corresponds to the empirical record that governance works best when it is locally contextualized and inclusive in terms of local practices and personal engagement of people, as seminally elaborated by Elinor Ostrom, and many others. As we have argued, this can be systematically grounded in a wider range of theoretical resources which have not yet been arranged together systematically. The second pillar of NBG is to tear down the walls of speciesism in thinking about institutions and to develop forms of rule-based multi-species interaction in which non-humans enjoy recognition, voice and representation. We have claimed that ritual is the paradigmatic form.

In the discourses about the roles of indigeneity in environmental governance, one approach is to recognize and reinstate Indigenous rituals and meanings in current practices ( Bush *et al.*, 2023). However, this would unduly constrain the view on ritual. The first step is to acknowledge the ubiquity of ritual across all societies, including Western late modernity: Just think of the rituals devoted to celebrities or sports events, political rituals, or the rituals of consumption, such as the public releases of the latest digital device. This acknowledgment triggers the switch of the Necker cube from disembodied institutions to embodied rituals as a generic and universal form of governance.

The question is how we can employ ritual in the practice of environmental governance, as we sketched in our NBS examples. Ritual is often associated with religious and quasi-religious stances, which we cannot imagine becoming a ‘policy tool.’ As said, one way is to tie up with existing traditions, which can offer rich inspiration, such as mentioned, Japanese Shinto. Another example is Chinese Fengshui that can inspire landscape design ( Yu, 2019). We generalize over these examples in referring to ‘place shaping’ as a distinct process that can nurture ritualization of reciprocity between people and nature, since eventually all ecological relationships are local in nature ( Grenni *et al.*, 2020). Landscape is a distinct notion that merges human subjectivities with ecological materialities ( Waldheim, 2016), and many human activities in landscapes (such as just walking) offer the potential for ritualization, such as inspiring concerns about nature and the eventual readiness to assume active roles, stewardships.

In principle, ritual can be seen as an object of design, such as deliberately creating a secular ritual for a specific purpose (the welcoming of newborn sheep is an example) ( Gordon-Lennox, 2017). However, there is no easy road to convincing people to follow a newly promoted ritual in terms of habitualization and embodiment. One powerful approach to ritualization is creating ritual affordances by means of artful design of the environment in which actions unfold. This stands in line with the concept of sensory governance. In fact, the instrumentalization of aesthetics is standard lore in modern business and marketing. Often, the problem is that the everyday aesthetics that emerge as a societal practice even block ecologically sensible practices (such as tastes for greenery that impoverish biodiversity) ( Saito, 2007). Hence, rethinking the material design of the human environment is strongly complementary to activating ritual as NBG: NBG downplays rational governance but highlights aesthetic, that is, sensory governance.

Art is well recognized as a tool in promoting eco-social transformation, mainly as a means to create awareness, to mobilize and to inspire ( Pearson *et al.,* 2018). In NBG, artful design becomes the key means to govern behaviour ( Herrmann-Pillath *et al.*, 2023). Art creates ritual affordances that can have dual functions, in the sense of semiotic co-option ( Maran, 2020), that is, multiple forms of interpretation in humans and non-humans, such that spontaneous coordination of interactions and eventually ritualization emerges. A simple and well-known example is to adapt features of buildings to the needs of birds, such as adding birdhouses, or paying attention to architectural features that allow for resting, bathing and so on. Once the presence of birds is widely recognized, a potential for ritualization emerges, such as welcoming migratory birds returning to the place.

Therefore, a promising approach to activating ritual for NBG is artful design of the material environment, including the human-built, and less the invention of new ritual practices. This is similar to the behavioural economics approach to nudging and recognizes forms of distributed agency unfolding in ritual practices. Artful design creates ritual affordances, and which specific practices emerge, is left to more-than-human creative responses.

## Ethical approval and consent

Ethical approval and consent were not required.

## Data Availability

No data are associated with this article.
