# Rituals as Nature-Based Governance of reciprocity between people and nature

**DOI:** 10.12688/openreseurope.17206.2

**Published:** 2024-08-07

**Authors:** Carsten Herrmann-Pillath

**Affiliations:** 1 Max Weber Centre for Advanced Cultural and Social Studies, University of Erfurt, Erfurt, Thuringia, Germany

**Keywords:** ritual as nature-based governance; interchange between people and nature; possession as more-than-human ritual; ritual governance of nature-based solutions

## Abstract

The conventional approach to environmental governance, based on institutions, regulations, and interventions, has failed to stop the current ecological catastrophe. I suggest a radical alternative: Ritual as the core mode of ‘nature-based governance’ (NBG) that enacts deep and comprehensive reciprocity between people and nature. NBG grounds governance mechanisms in embodied more-than-human practices with normative force. I build on theories of embodiment to suggest a general concept of ritual that is inspired by but generalizes over Indigenous thought and is informed by East Asian ideas about ritual as the pivot of social order. Further, the embodiment framework recognises ritual as a kind of action humans and non-humans share as living beings. Therefore, rituals can be harnessed in workable governance mechanisms to create and sustain communities of multi-species cohabitation. I distinguish between two basic types of reciprocity corresponding to two types of governance: Disembodied reciprocity enacted by conventional human-only governance schemes and embodied reciprocity enacted by NBG. Embodied reciprocity creates relationality of people and nature. Equipped with these theoretical insights, I suggest practical applications in the context of NBG of Nature-based solutions, discussing three stylized models. These are the formation of urban multi-species communities in urban gardening and urban forests, the commoning of ecosystem services of animal populations in wildfire protection, and reconceptualizing eco-compensation as a reciprocal ritual of gift-giving,

## 1. Introduction: Ritual as a mode of governance

This paper in practical environmental philosophy defends the claim that ritual is an alternative mode of governance to governance by institutions (government, law, regulations, organizations) and offers a new approach to put the broken relationship between humans and nature back in order. This claim of novelty refers to what has been established as ‘mainstream’ in environmental policies and ties up with other more radical critiques, especially concerning the inclusion of other species in environmental policies and planning ( Houston *et al.*, 2018). As an economist by training, my critique of the mainstream argues from an insider position, building on systematic efforts to rework the fundamentals of that discipline to achieve an integration with advanced social theory, in particular regarding embodiment and materiality of human economic actors ( Basso & Herrmann-Pillath, 2024).

The paper is motivated by the empirical diagnosis that the conventional approach to environmental protection and policies has failed to meet our times' challenges. This is especially true for biodiversity ( Dasgupta & Levin, 2023; Hochkirch *et al.*, 2023; United Nations University - Institute for Environment and Human Security (UNU-EHS) *et al.*, 2023). Something is fundamentally wrong in our relationship with nature. The reason is that speciesism deeply permeates our modern worldview, and even most scientific thinking views the human species as being distinct in some essential respects from all other species ( Singer & Harari, 2023). This combines with anthropocentrism in approaching human action and policies worldwide, such as maintaining this position when analysing ecosystem services ( Dasgupta, 2021). The separateness of humans is grounded in ideas about the uniqueness of the human mind and culture, which not only downgrade the position of other species but also reinforce a Cartesian denigration and devaluation of the human body in ideas about mind and rationality ( Johnson, 2017). After all, it is ‘being a body’ (and not ‘having’), which is a fundamental feature that is shared with all other species ( Merleau-Ponty, 2021), and the paradigm of dominating other species goes hand in hand with the idea of governing the human body by rational thought and rational institutions ( Foucault *et al.*, 2004).

I outline a new approach to governance, ‘Nature-based governance’ (NBG), in which ritual is a pivotal concept. The terminological innovation of NBG was suggested by members of the COEVOLVERS project (co-evolvers.eu; Beskydy team, led by Tatiana Kluvankova) when preparing a workshop on Earth System Governance about governance on nature-based solutions ( https://www.earthsystemgovernance.org/news/2024-beskydy-workshop-call-for-papers/). I expand its meaning substantially, on par with other approaches to governance labelled by specific epithets, such as ‘adaptive’ or ‘global’ ( Bennett & Satterfield, 2018). ‘Nature-based’ in this paper approaches humans as part of nature, while recognising that in the transition to the Anthropocene, the human domain has expanded to effectively include the biosphere in its range, obliterating the division between nature and the human domain, especially including human-built artefacts ( Herrmann-Pillath *et al.*, 2022; Vogel, 2015). I do not separate the human domain in terms of ‘non-natural’ institutions in governance, apparently defining its autonomy from nature. On the contrary, I claim that ritual is an institutional form that transcends the human/non-human divide and, therefore, can govern human behaviour as a critical determinant of ecosystem performance and evolution. Traditional forms of environmental governance aim to govern human behaviour concerning nature as an object; NBG conceives of governance architectures as ‘institutional natureculture’, matching with the materiality of natureculture assemblages in which humans and other species co-habit ( Malone & Ovenden, 2016).

Ritual is the foundational analytical category that breaks through speciesist constraints in our thinking about a more-than-human community, thus becoming a central, though still neglected category in posthuman social thought ( Braidotti, 2019). Ordering interaction between living beings by ritual is a biological universal ( Rossano, 2016; Stephenson, 2015). Hence, ritual is the obvious candidate if we search for a most general category that could apply to governing people’s relationship with nature. Whereas we cannot conceive of law as being shared between human and non-human species, ritual is one category that applies to both. There are others, such as play ( Merritt, 2021); but I leave this for other work. However, ritual relates to many of those, for example, since play often includes ritualised performances. The same is true for art since ritual is also an aesthetic practice ( Prum, 2017).

Nature-based governance receives inspiration from Indigenous ways of life, often referred to in the current literature on radical eco-social transformation ( Alexander, 2013; Kimmerer, 2020). However, I go beyond this reference in asking the question of to which extent and how ritual can become a medium of NBG in modern postcapitalist societies, specifically in densely populated urban settings in which most humans will live in the future, especially in the Global South. The discourse on indigeneity is mainly shaped by the experience of formerly colonial societies where Indigenous people still form a distinct group with increasingly influential voices and a responsive and responsible academic community, such as North America, Australia, or New Zealand (for example, ( Salomon *et al.*, 2023). My foundational discussion of ritual combines this discourse with the literature on ritual in societies where ritual was and often still is a central force in governing society and has been theorised as such for millennia, foremostly in East Asia. Exclusive reliance on Indigeneity bears the risk that, as in the Rights of Nature movement, the focus is on nature that is relevant to their spirituality, such as recognising certain rivers in ontological relationality ( The Cyrus R. Vance Center, 2020), while leaving open how these integrative steps can be upscaled and transferred into entirely different cultural contexts ( Wesche, 2023). As experience shows, for example, in the context of the United Nations, this transfer can happen via ritual innovation, syncretism, and even secularisation, in which Indigenous rituals become universal forms of practice ( Chamel, 2022). Ritual innovation is a common feature of such practices ( Bell, 1997, p. 223ff) and can start from individual creativity ( Kimmerer, 2020, p. 249ff).

Ritual is almost synonymous with ordering behaviour and is, therefore, an essential form of governance, which is, however, neglected in established institutional approaches to governance, where it is mainly seen as cultural means to create legitimacy to institutions, such as conveying an aura of sanctity to a judge. This view is the consequence of the distinct cultural history of ritual in Western modernity ( Asad, 1993). The neglect of ritual in contemporary social thought reflects what Charles Taylor ( 2007) has called the ‘excarnation’ of Western societies in the aftermath of the Reformation, that is, the growing compartmentalisation between embodied and disembodied spheres of life, with the disembodied spheres being the domains of rationality (bureaucracy, market, science, etc.). This view is mirrored in Max Weber’s influential approach to modernisation as rationalisation. Tellingly, Weber struggled with understanding the role of ritual in China, which he disqualified as ‘enchantment’ and the antipode of Western rationality as ’disenchantment’ ( Feuchtwang, 2010; Yang, 2020). For him, Confucian ritualism was a major reason China did not transform into capitalism, even though it had appeared as the most advanced stage of civilisation to Enlightenment thinkers such as Wolff, Quesnay and Voltaire. Considering Indigenous ritual as the main reference for discussing eco-social transformation would exclude many societies in which ritual has been a dominant mode of ordering society and is still influential today, such as China ( Herrmann-Pillath, 2016a) or Japan ( Kawano, 2005; Nelson, 1992). For example, the Shinto ritual has inspired local projects of eco-social landscape restoration of traditional satoyama agricultural commons ( Berglund *et al.*, 2014; Ishizawa, 2018) and community revitalization with renewable energy projects ( Hiroi & Caspary, 2024). In China, Fengshui rituals have inspired urban landscape design in megacities, such as in the context of the sponge city as an assemblage of NBS ( Yu, 2019).

Beyond foundational work, the paper refers to Nature-based solutions as a workhorse for discussing practical implications. NBS are widely regarded as a powerful means to meet the challenges of climate change in urban societies ( Bush *et al.*, 2023; Hobbie & Grimm, 2020; Kabisch *et al.*, 2017), increasingly also considering the Global South beyond the mainstream cases in regions such as Europe or Australia ( Diep *et al.*, 2022). Governance issues are critical in arranging NBS, making them sustainable, and scaling-up ( Albert *et al.*, 2019; Sarabi *et al.*, 2020). NBS are mostly conceived as contributing to goals defined by the needs of human societies to which the service is rendered ( Gomez-Baggethun, 2018). Protecting and nurturing the ecosystem is a mere functional requirement of sustaining the capacities for generating these services (‘natural capital’ ( Bateman & Mace, 2020)). However, there is also the idea of a reciprocal relationship between people benefitting from nature and nature itself, mostly conceived in abstract terms as sustaining biodiversity by NBS design ( IUCN, 2020). However, this is merely a standard for assessing the performance of an NBS, and the notion of reciprocity is devoid of any substantial meaning in terms of the type of interaction ( European Commission, 2021). This is where the notion of ritual comes into play since reciprocity is a term thoroughly explored in anthropology, where it is often seen as being ritually performed, such as in rituals of gift-giving (for an East Asian example, ( Rupp, 2003).

I posit that NBG conceptualises reciprocity rigorously, and not just metaphorically, as an embodied flow of gifts between nature and people governed by rituals. This follows the growing awareness that the relationship between nature and people must be reciprocal ( Ojeda *et al.*, 2022). This idea is often associated with Indigenous thought ( Kimmerer, 2020, p. 380ff) and has been stated as a general requirement in designing Nature-based solutions as distinct from ecosystem services ( Maller, 2021). Hence, I claim that NBG theory can contribute to rethinking NBS as designed for and with nature, and not just using nature for human goals ( Seddon *et al.*, 2021).

In the following, Section 2 starts out by discussing the two pivotal concepts, ritual and governance. The section continues distinguishing between two basic forms of reciprocity, embodied and disembodied, which relate to alternative forms of governance. Ritual enacts embodied reciprocity as a form of relationality. I illustrate this theoretical argument by discussing the relationship between the territoriality of living beings and human ways of relating to land: disembodied, as in Western modernity, and embodied, as in Indigeneity. Section 3 applies the argument to three stylised cases of NBS that correspond to real-world Living Labs in the COEVOLVERS project, where I champion ritual as a new approach to governing NBS. Section 4 concludes with thoughts on art as a medium to create secular rituals in urban societies.

## 2. Fundamentals of NBG

### 2.1. Defining ritual

Let us preliminarily define ‘ritual’ as a specific type of practice ( Bell, 1997; Hobson *et al.*, 2018). Ritual is a regularised behavioural pattern that is recurrent through time, however, with no fixed schedule, often triggered by certain events which are themselves determined by the ritual. I refer to this as ritual affordances (including calendar rites, biological rhythms, types of encounters, or life-cycle events). There are individual rituals (such as those related to cleaning and hygiene) and collective ones, which include a wide range of types of interaction. In the case of humans, ritual affordances often include artefacts specifically designed for that purpose. A key feature of ritual is that the regularity is deeply embodied; there is only marginal engagement of rational reflection and other choice mechanisms. Ritual is a flow of pure action. However, this does not mean there is no awareness, as it would apply to purely automatic behaviour as a type distinct from ritual. An important aspect of human ritual is the sincere deployment of the ritual practice and less the cognitive states accompanying the actions ( Seligman *et al.*, 2008). In other words, the specific reasons why ritual is deployed are not essential but proper and exact enactment of ritual (orthopraxy as opposed to orthodoxy). This critical role of proper doing the ritual is the key to establishing ritual as a concept that applies uniformly across humans and non-humans. Finally, the importance of ritual for discussing environmental policies stems from the fact that ritual is deeply performative: Rituals enact worlds and have ontological powers ( Mc Graw, 2015), hence can trigger and sustain processes of ‘becoming world’ ( Houston *et al.*, 2018).

Ritual ties up with critical notions such as embodiment, distributed agency, or habitus in playing an essential role in forming human communities and the identities of their members, grounded in emotional attachments and shared practices of expressing them. The influential Durkheimian view sees ritual as the cement of community, especially in religion ( Durkheim, 2012). Goffman represents another line of thinking that explores the role of rituals in everyday life and even apparently minor encounters ( Goffman, 1982). One recent approach integrating these views is Collins’ theory, in which rituals are fundamental in ordering social interactions by mobilising and sharing emotional states among actors ( Collins, 2005). Like with a Necker cube, Collins switches the view on institutions such as the market from the established emphasis on rational design and choice to the view on ritual. His approach implies that we can envisage a frameshift from institutions to rituals across all domains of human sociality. Once this step is done, ritual can also expand the theory of governance to the more-than-human.

In the Durkheimian view, ritual relates to the sacred. This seems different from Goffman’s approach to everyday rituals; however, as the East Asian framing suggests ( Nelson, 1992), the question is what happens if a ritual were not conducted. Ritual differs from habits or routines in what happens if these practices are not enacted ( Seligman *et al.*, 2008). Take, for example, a greeting ritual that may appear to be a mere routine. However, if it is not enacted, this opens an abyss of negative potentialities, as this neglect could only be interpreted as intentional and signals a breakdown of relationships, mistrust and disorder. In comparison, if I keep the habit of always wearing a hat when going outside, people may wonder when I leave without it but do not feel that disorder threats. If a family has routines for preparing breakfast, deviating from them may create friction in arranging things properly and swiftly, but not expressing a caring morning greeting may reveal a deeper issue. In sum, ritual is about what people value as sacred in the sense of being non-negotiable and fundamental to their lives.

The distinct role of values has been well recognised in ecological and sustainability sciences ( Intergovernmental Science-Policy Platform on Biodiversity and Ecosystem Services, IPBES, 2022). When considering ecosystem services, values take different shapes, such as instrumental values. In this case, humans assess the functions of a natural entity in meeting goals defined by humans, such as considering the functions of trees in regulating urban microclimate and water flows ( Behm *et al.*, 2022). Governance schemes focus on creating the technical and social conditions for properly fulfilling these functions. In contrast, intrinsic values ascribe non-transactional values to nature independent from human goals, in the Kantian sense of a purpose-in-itself or non-negotiable and incommensurable dignity. However, this creates a quandary: are these values intrinsic to nature, or are they ultimately values that humans have and only ascribe to nature? If humans do not have these values, how can we imbue their thinking and feelings with such values if we deem them necessary for repairing our relationship with nature? These questions point to governance as a solution; however, whereas standard governance schemes are outcome-oriented in that certain functions should be realised by the actions under their scope, we are now asking for a governance scheme that creates values intrinsic to the relationship between humans and nature. This scheme is ritual.

### 2.2. Governance

In environmental policies, governance refers to all institutional and administrative means that enforce policies and laws intended to resolve environmental dysfunctions in the economy and society ( Young, 2013). The most general meaning of governance is enforcing and monitoring the ‘rules of the game’. This definition leaves open the question of who enforces and monitors. Diverging answers is one reason why different disciplines and fields of application give different meanings to the terms on a more specific level. One important strand is to approach governance as a form of government that transcends conventional hierarchical-bureaucratic modes and activates societal forces and groups to achieve public goals ( Treib *et al.*, 2007). Another strand conceives governance in more articulate opposition to government and as a form of social self-organisation, especially in community governance. Further, notions of governance may emphasise different aspects, in particular, efficiency versus equity ( Bennett & Satterfield, 2018): The former emphasises performance, effectiveness, and resilience, and the latter emphasises justice, inclusion, and democracy.

The literature on governance often approaches the term as if it implicitly has an independent theoretical status, in the sense of a 'theory of governance,' which differentiates into various divisions such as domains or modes of governance. However, such approaches tend to leave behavioural foundations out of sight and approach governance only in terms of structures, institutions, and mechanisms, such as bureaucracy, networks, or markets ( Pahl-Wostl, 2019). This incompleteness may explain why economics often lurks behind theories of governance since economics offers an integrated view of behaviour and institutions.

Economic paradigms have strongly influenced the established governance theory in that institutions are often treated analytically independently from actors who respond rationally to institutionalised incentives ( Manheim & Spackman, 2022; Williamson, 1985). These can be modulated by cognitive factors such as worldviews and ideologies ( North, 2005). Economic theories count in the context of ecology because most ecological challenges result from economic action and, therefore, require economic governance mechanisms that are at least complementary to others. However, this also creates conceptual tensions when combined with other approaches to governance that emphasise community aspects, inclusion, or care. What needs to be improved is an integrative theory of governance since otherwise, practices of governance fail because the different approaches rest on fundamentally conflicting anthropological and behavioural assumptions.

Institutional economics distinguishes between formal and informal institutions, which is also essential in governance. Formal institutions include environmental law and administrative measures to enforce and monitor it. This needs to be done not only by governmental institutions but also by measures such as voluntary agreements on labelling eco-friendly products, which are enforced and monitored by private-sector agencies. Informal institutions include various forms of governance in social groups, such as observing rules of conduct of group members, for example, neighbourhoods or peer groups. A question is how far internalised norms are included in these notions. For example, the socialisation of children may proceed in a formalised institutional setting, but eventually, the norms become internalised values that later are no longer formally enforced.

This observation alerts us of the significant difficulty that results from neglecting the behavioural foundations of governance. Economics assumes rational actors, which implies parsimonious incentive-based approaches to understanding and designing governance. However, if people are value-driven, emotionally inspired, or altruistic, the implications for governance would differ widely. To add complications, we know that governance mechanisms are performative in that a particular type of governance may also generate a particular type of actor, contrary to the universalist claims of rational choice paradigms ( Bowles & Polanía-Reyes, 2012; Herrmann-Pillath, 2016b).

These issues have been highlighted in recent developments in governance theory that view behaviour as emerging from assemblages of actors and artefacts, particularly in research on infrastructure. In discussing NBS against the previously invoked background of the collapsing border between nature and culture, the concept of ‘green infrastructure’ looms large, which allows to establish a direct methodological linkage between conventional governance approaches and alternatives that regard nature itself as an integral part of governance ( Gomes Sant’Anna *et al.*, 2023). Infrastructure is ambivalent in that it mainly remains invisible and channels behaviour via material constraints and affordances, such as using tap water without people being aware of the vast and complex infrastructure enabling water flow ( Star, 1999). However, at the same time, infrastructure can become highly politicised and, hence, visible ( Larkin, 2013). The invisibility of infrastructure relates to forms of governance that mainly operate via embodied affordances. This observation has motivated the creation of new governance concepts, such as ‘sensory governance’, where governance operates via material signs in the artificial environment without invoking formal regulations and incentives ( Schulte-Römer, 2023). This concept is close to the nudging approach as a policy tool designed by behavioural economists ( Thaler & Sunstein, 2021). Infrastructure aligns with embodied rationality emerging in assemblages of actors and infrastructural artefacts ( Manheim & Spackman, 2022).

Where do we locate ritual in this theoretical canvas? Two observations need attention. The first is that ritual does not easily fit into the categorisations of institutions because all aspects count in a complex way. First, many rituals are formalised, often in great detail, and some bodies monitor and enforce proper rituals, such as in churches as a form of religious community. However, this is not a necessary feature of ritual, as second, ritual is often observed in Indigenous communities without external enforcement, but violating ritual expectations may trigger strong social sanctions, including ostracism. Third, ritual is internalised chiefly because proper adherence to ritual is an essential component of individual identity as a group member, which also grounds spontaneous group sanctions of improper behaviour. These three points show that ritual is a phenomenon sui generis, crosscutting the analytical distinctions in standard institutional approaches to governance. The analytical distinction between the actors and the institution becomes obsolete in ritual.

The second observation is that institutions are widely conceived as distinctly human. We would not count behavioural regularities in baboon troops as 'institutions’ and approach hierarchies in those groups as 'governance structures'. There are various reasons. One is that human institutions appear to be culturally contingent, hence variable independent from genetic factors, whereas non-human regularities are seen as genetically determined. Another reason is that institutions are mediated mainly by language, conceived distinctly human ( Searle, 2011). In this sense, governance could not be 'nature-based.' This view is a problem regarding environmental issues since established uses of 'environmental governance' tend to include the ecosystem when assessing governance performance but exclude the non-human actors from the reference domain of 'governance'.

### 2.3. NBG and reciprocity

If we combine the two observations, we get the inspiration that ritual may be an alternative form of governance not affected by the claims of human distinctiveness related to institutions. Whether we do so depends on how we further dissect the notion of ritual and which weight we give to the second and third points above: the role of social sanctions and internalisation in ritual governance. Following the introduction, this relates to the more fundamental question of to which extent embodiment grounds governance, more-than-human. This approach is 'Nature-based governance':

*Nature-based governance grounds governance mechanisms in embodied more-than-human practices with normative force.*

A line of thinking relates ritual to biological roots as a deeply embodied form of human behaviour that enables the distinctly human form of ultrasociality ( Rossano, 2016). Ritualisation is a primary form of behavioural coordination in animals that is genetically endowed, but in terms of activation shaped by epigenetic mechanisms, such as learning by imprinting that allows for contextualisation beyond genetic fixation. A standard example is the ritualisation of birdsong, which is genetically endowed but learned in a specific population context to show great intraspecies variety across local bird habitats. Birdsong is a medium that literally orchestrates intra- and interspecies cohabitation in a specific territory ( Despret, 2019). In this sense, ritual is a coevolutionary phenomenon that leaves much leeway for forms of behaviour which transcend genetic determination and converge to human forms of culture, such as in aesthetic practices ( Prum, 2017).

In the following, I will focus on one specific aspect of ritual: ritual and reciprocity. As said, reciprocity has been emphasised in recent debates about NBS. The literature on reciprocity is vast and has accumulated over decades in anthropology, the social sciences and biology. However, the embodiment aspect has not been systematically explored, even though at least two strands point towards its central significance. One is the biological literature on kin selection; the other is the anthropological literature on the gift. I suggest distinguishing two types of reciprocity: embodied and disembodied.

- Disembodied reciprocity relates to the common conceptions of governance, with the market as an archetype. In this case, humans and other species are seen as separate entities that enter a reciprocal, transactional relationship, such as considering the ecosystem services of urban greenery for humans on the one hand and developing measures enhancing biodiversity on the other hand (for example, ( Connop *et al.*, 2016). This approach does not require that the humans that enjoy benefits from ecosystem services also enter a relationship with the other species that receive benefits in turn, even to the extent that citizens may not be aware of the reciprocal transactions which may be enacted by specific government agencies. Disembodied reciprocity is mediated by abstract media such as money and formal regulations that quantify the mutual flows, such as carbon offsets in ecocompensation ( Wang *et al.*, 2022).
- Embodied reciprocity establishes relationality between the two beings who act reciprocally ( Eyster *et al.*, 2023). That means, in the strict meaning of a gift, ecosystem services and biodiversity enhancement are coupled via the relationality of the two beneficiaries, which is embodied and situated ( Trosper, 2022, p. 49ff). Media of embodied reciprocity are actions that engage embodied sensory experiences, such as touch or sound, and that are placed in the ecosystem context in which both recipients live. The governance format that enacts embodied reciprocity is ritual. Correspondingly, scientific accounts of embodied reciprocity rely on non-quantitative data such as narratives ( Kimmerer, 2020; Maran, 2020)

In both cases, reciprocity can be a set of single actions that are not sustained through time and that do not upscale. The critical function of governance is perpetuating such actions and stabilising reciprocity regimes. A disembodied scheme of ecocompensation is sustained via formal regulations and incentives that define the course of actions and enable anyone to partake. In contrast, ritual is an embodied scheme of governance sustained via chains of recurrent ritual procedures and expansive in terms of the community of people who join the ritual practices.

Ritual is a relational category in many senses, such as relating ritual objects and people or relating ritual practitioners in a group enacting the ritual. I suggest neatly distinguishing between ritual and relationality as used in positing indigeneity as a benchmark for designing NBS. Ritual is a form of governance that guides behaviour enacting relationality. Relationality is a cognitive and emotional state in which assemblages of beings are created who experience themselves as related via embodied media of ‘intra-action’, to use Barad’s term ( Barad, 2007). For example, a ritual devoted to a certain tree may create a relational assemblage of tree and humans enacting the ritual, in which the humans experience this relatedness as an embodied state, consciously accessible via feelings and emotions, and, depending on cultural context, also in referring to the tree as a partner, such as in Indigenous spirituality, but also a modern urban context, as the famous case of Melbourne citizens sending emails to trees demonstrates ( Gulsrud *et al.*, 2018).

A case in point that plays a critical role in discourses about indigeneity is the relationship between territoriality and land and the related forms of governance ( Boydell, 2010; Wagner & Talakai, 2007). In Western modernity, sovereignty and property are the key institutions of governing land. As has been intensively documented, this includes governing the exclusive land assignment to groups and individuals by means of systematic mapping. Mapping has been a critical means of colonial dispossession and of subjecting land to a process of disembodiment, that is, imposing a regime of extracting ecosystem services in a compressed range of economic uses defined by abstract systems of measuring economic performance ( Bhandar, 2018; Vanuxem, 2022). Accordingly, maps reduce land to a mere object of property rights assigned by sovereign authorities. In contrast, Indigenous subversive mapping aims to record the flow of embodied interactions with the land that express and enact the relationality between people and other beings cohabitating on the land ( Chao, 2022).

It is illuminating that there are two ways to govern the relationship between land and people that would both recognise cohabitation. The disembodied form would establish disembodied governance by assigning formal property rights to other species ( Bradshaw, 2020; Hadley, 2015). Some protagonists of animal property point out that this is rooted in biological universals of claiming and controlling resources, most salient in territoriality, which bears many resemblances with human claims on land. Apparently, in a paradoxical way, this view aims at healing the relationship between humans and nature by including other species in the very formal system of governance that until today has been employed to enforce human exploitation of nature, and even claiming that this system of governance is rooted in biological universals ( Gintis, 2007). As said, in the history of land ownership, there was a transition towards regimes in which land is conceived as an abstract parcel on a map that defines the boundaries and identified proprietors: land registries ( Vanuxem, 2022). These institutional innovations deliberately targeted local property institutions such as the commons, which were community-based and rooted in traditional norms of land use ( de Moor, 2018). Since these community norms were enacted in various customary practices, we can conceive them as forms of ritual governance, the second way of governing the relationship between land and people. Ritual governance reveals the role of non-human signals in expressing possession, referring to all kinds of ritualisation of territoriality without establishing the exclusiveness of human formal property ( Despret, 2019; Gibson, 2021; Herrmann-Pillath, 2023). Hence, it is not only straightforward to recognise the possession of non-humans but also to understand ritual as the universal form of claiming possession among humans and non-humans. There is a wider range of linguistic and non-linguistic performative behaviours, how possession is recognised and stabilised among human actors without invoking the law ( Rose, 2019).

In this context, the debate about the legal status of Indigenous claims on land is highly significant. Indigenous lawyers strongly emphasise the radical difference between common law notions of property (this discussion mainly unfolds in former British colonies) and the Indigenous relationship with the land ( McNeil, 2017). The latter emphasises the shared identity of people and land, even to the degree of seeing landscapes as embodiments of hybrid beings, thus blurring the demarcation between the living and the non-living ( Povinelli, 2016). One of the critical distinctions is the inalienability of the land as a sacred homeland, which straightforwardly can be interpreted as a difference between ritual and institutional governance of land and defines a relational understanding of possession ( Keenan, 2015; Trosper, 2022, p. 85ff). These Indigenous views also recognise the shared ownership of resources by humans and non-humans: This is a paradigmatic case of NBG by ritual—the relationship with land grounds reciprocity between people and nature that is ritually enacted.

## 4. Practical significance: Ritual in Nature-Based Solutions

Indigenous ritual reflects the past and present closeness of Indigenous ways of life to nature. This closeness can sometimes also be found and re-enacted in places with an ecological richness and vast spatial resources, such as North America or New Zealand. However, most humans will live in dense urban settings where nature has been downgraded to a mere urban amenity, such as public parks. Grey infrastructure is dominant. This does not stop cities such as Singapore from embarking on a strategy for rewilding the city, which requires inventing and sustaining genuine natureculture assemblages that combine green and grey infrastructure ( Apfelbeck *et al.*, 2020; World Economic Forum, 2022). As we know from experience, governance is a critical issue, as there are many obstacles to implementing such measures and, most importantly, upscaling them ( Scolobig *et al.*, 2023). This is often seen as an issue relating to the directly engaged agencies. Still, we also know that many NBS cannot be performed without the direct engagement of citizens ( Brokking *et al.*, 2021; Frantzeskaki, 2019).

Suppose we conceptualize the relationship between people and nature in terms of reciprocity. In that case, the ritual approach suggests the most general notion of gift exchange: In the context of NBS, the ecosystem service activated in the NBS would be conceived as a gift given and not just a service rendered, which creates the obligation to give a gift back in return. Gift-giving differs fundamentally from other types of exchange, mainly market exchange, as the gift-giving enacts an enduring relationship between the two sides. That means implementing an NBS amounts to ritually creating an ecosystem community; hence, it is one of the essential forms of commoning ( Leitheiser *et al.*, 2022).

The first model I discuss is the one closest to the Indigenous benchmark, namely urban gardening. This is relatively easy to conceptualise ritually, as the relationship between people and nature is direct. However, this does not apply to the neighbourhood where the garden is located and might even be excluded or entirely disinterested in the garden. In this case, ritual governance can expand the flows of embodied reciprocity to include both gardeners and neighbours. In the second case, wildfire protection by animals, the relationship between herders and neighbours is mainly instrumental and indirect, even to the extent that neighbours may feel encumbered by the presence of sheep. I argue that ritual is a governance scheme that expands the stakeholder analysis of governance to include non-humans by establishing a direct relationship between humans as stakeholders of wildfire protection and the animals as stakeholders of the habitat. My third case is the hard one, namely greening grey infrastructure that can even be seen as an antipode to nature, such as parking lots. However, this is also the most typical case compared to other cases, especially in large urban conglomerations.

### 4.1. Urban gardening

Urban gardening has a rich and diverse tradition in many forms, such as parks, community gardens, or household plots. Gardening can be seen as NBS in various functional contexts, such as stabilising urban microclimate or contributing to public health ( Camps-Calvet *et al.*, 2016; Sowińska-Świerkosz *et al.*, 2021). There is a wide scope of formats for urban gardening which can activate even marginal spaces such as verges between roads and housing plots ( Kingsley *et al.*, 2021). In my discussion, I include the role of urban forests and other forms of renaturing cities, which can also be realised in different forms, such as being planned and implemented by municipal authorities or created by citizen activists as a community endeavour ( Dushkova & Haase, 2020). Urban forests and urban gardens can be spatially integrated micro-ecosystems with many forms of hybridisation, such as fruit trees and berry bushes.

**Figure 1.**
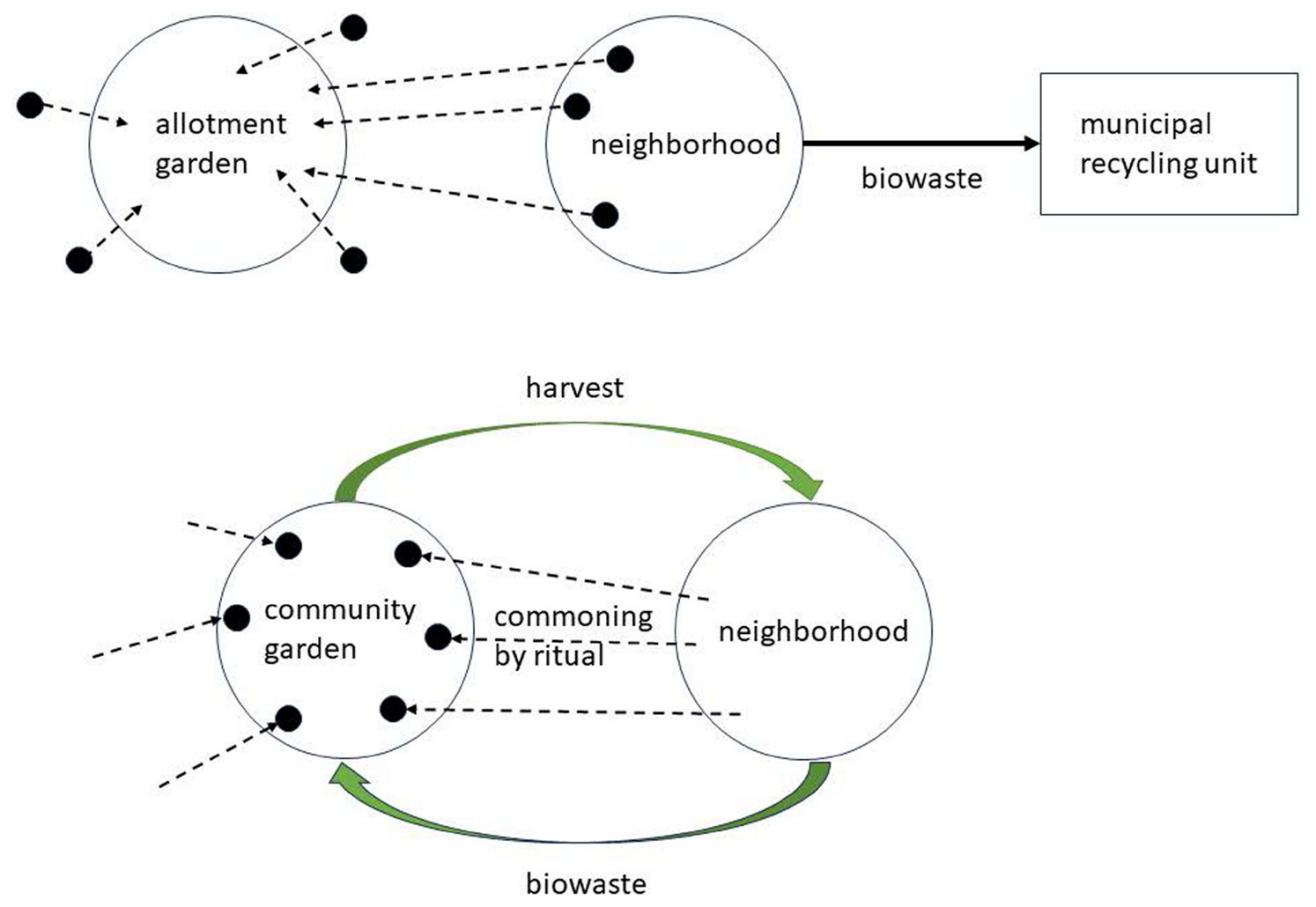
Two forms of urban gardening.

Let us consider one variant: a community garden maintained by a local group of human hobbyists. Community gardens can take different institutional shapes, such as allotment gardens or collectively managed schemes. Community gardens claim valuable urban space and are always potentially threatened by urban development. An alleged solution is to implement an eco-compensation discussed in Section 4.3. Often, this is endorsed even by the neighbourhood since the garden users may be a minority or even partly outsiders ( Figure 1, top), which is especially true for allotment gardens, where the beneficiaries of the institution are exclusively the individual holders of lots. In contrast, the ritual approach to commoning creates a setting of mutual flows of reciprocal gifts between the various parties of the local ecosystem. It emphasizes the collective management of the garden.

One example of the potential for ritualization is the essential role of composting, which needs the input of organic wastes ( Puig de la Bellacasa, 2017). A principle of modern waste management is to make waste invisible to urbanites; hence, even when it comes to organic waste, it is collected in bins and transported to industrial composting sites ( Figure 1, top).

This is a case of disembodied governance of reciprocity where biowaste is given back to nature. In turn, nature gives food resources, with most people in the neighbourhood lacking any direct experience with doing agriculture and gardening. People are not engaged in this form of disembodied reciprocity; on the contrary, the less ecologically minded may not even see the connection between food and recycled biowaste; they discard the waste following certain regulations and admonishments from authorities. In the urban garden, hobbyists mostly may use biowaste for composting, but this remains limited to the direct users of the garden. There is no embedding of the garden in the community of the neighbourhood, which makes it vulnerable to redevelopment initiatives.

The ritual approach suggests creating flows of gifts between the garden and the neighbourhood, where the latter sends the gift of organic waste to the garden, and the garden returns gifts, such as vegetables and fruits, for example, at certain communal festive events ( Figure 1, bottom). The flow of gifts connects the community garden to the neighbourhood, which is independent of the actual engagement of all residents in the garden. Gardens can be rooted in the community in many other ways, such as beekeeping. Even for the bees, a similar mechanism of creating ritual relationships is feasible as for the previous case of the sheep, that is, individualizing bees by tagging and making them visible to neighbours as individual visitors to their balconies ( Chittka, 2024) https://www.pollinatinglondontogether.com/. In sum, rituals of composting create relationality between people and nature and people and people: In embodied actions of reciprocity, people collect biowaste as a contribution to composting and later experience nature giving back in the form of the harvest. The point of the ritual is that this does not require meeting standards of disembodied reciprocity: The harvest must not match the effort of collecting the waste. Ritual symbolism elevates even a single salad enjoyed at the community festival to the embodied experience of gift exchange with nature.

Gardening is a crucial example, if not a paradigm for nature-based governance: A human gardener must pay attention to the natural conditions and requirements of the garden and hence is 'governed' by its rules ( Schwarz, 2023). Without giving back and caring, the garden cannot flourish. Composting is a vital activity that is made invisible in what has been aptly referred to as 'sensory governance' in urban infrastructure ( Schulte-Römer, 2023), as in this case, rendering waste invisible and separated from the living eco-community, staying in the long tradition of urban governmentality for public order and sanitary improvements. Making organic fertilizing possible via ritual flows of biowaste gifts changes this pattern of sensory governance in raising awareness among all residents about the connectivities in the local ecosystem.

### 4.2. Wildfire protection

**Figure 2.**
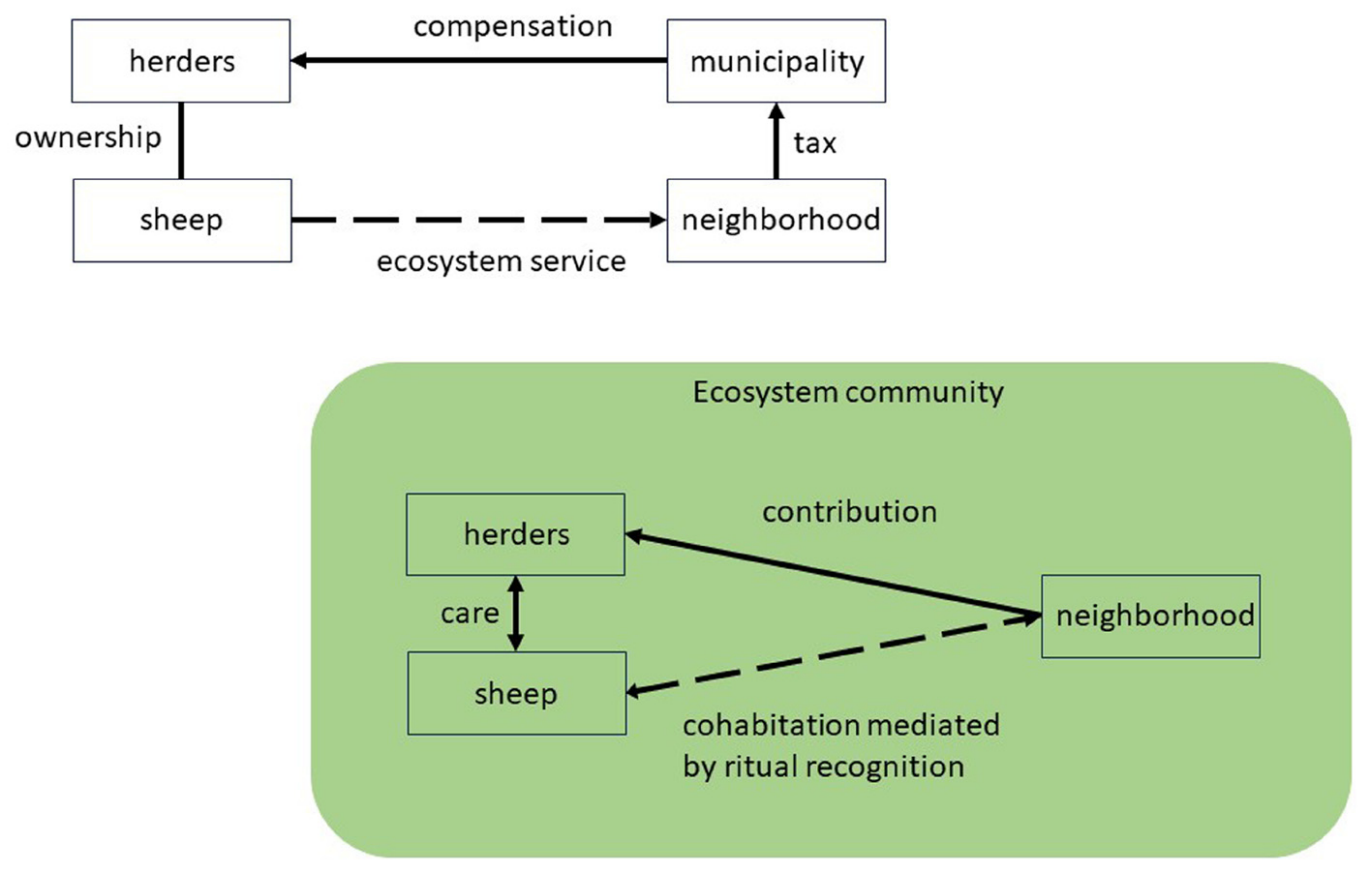
Wildfire protection as ecosystem service versus ritual governance of common good.

The second example of the ritual approach to NBS is using animals in wildfire protection, such as sheep grazing in areas bordering forests and human settlements ( Ribeiro *et al.*, 2023). A standard approach views this as a problem of compensating herders for rendering the service to keep, monitor, and protect the sheep. That means the regulatory arrangement is only among humans and does not include the non-humans as stakeholders or parties. For example, the neighborhood may pay a fee to the municipality that arranges the service managed by herders ( Figure 2, top). There is no relationship between people in the neighbourhood and the sheep who generate the ecosystem service of grazing. On the contrary, sheep may often be counted as a nuisance.

In this stylised case, we can connect to the recent literature on stakeholder analysis of NBS, which is mostly informed by disembodied theories in the management and social sciences ( Reed *et al.*, 2009; Zingraff-Hamed *et al.*, 2020). The standard question is how various stakeholders in a scheme, such as wildfire protection, can be identified, how they can be included via participatory mechanisms, and how incentives can be created that engage them in sustaining the NBS. However, participatory mechanisms exclude other species, in the current case, even the service producers, because they rely on language as a key medium. Therefore, stakeholder analysis has recently been extended to include other species, such as via mechanisms of recognition and representation ( Kortetmäki *et al.*, 2023). The difficulty remains in how the interests of non-human stakeholders can be made commensurable with the standard measure of stakes in the human domain, and how their interests can be communicated to humans ( Hernandez-Santin *et al.*, 2023).

The ritual perspective suggests the radical shift to regard the animals as members of the NBS arrangement with full rights (citizens in the sense of ( Donaldson & Kymlicka, 2013) who discuss the imaginary case of "Sheepville" (pp. 135ff). I In principle, administrators may recognize the sheep in institutional terms by assigning legal status to them and representing them by human stewards. However, the relationship quality between people in the neighbourhood and animals would remain the same. Ritual is transformative as it would enact a process of commoning, building, and sustaining a community of sheep and humans, especially those in the urban neighbourhood who ultimately benefit from the ecosystem service. From the economic standpoint, all community members benefit from a common good: wildfire protection. Commoning enacts this mutual sharing of benefits as another form of gift exchange, which includes transforming the fee in the regulatory approach into a contribution paid directly to the herders for their engagement ( Figure 2, bottom). However, ritual adds many aspects of immediate recognition of animals as members of the local ecosystem community, which includes the forest, the border area, and the urban neighbourhood.

For example, rituals include all forms of story-telling that express a relationship between humans and animals or all variants of direct mutual engagement of humans and animals. An example is practicing the welcoming of newly born members of the community: Humans may celebrate the birth of sheep and recognize their individuality as members of the community, for example, by giving them names. Settings of ritual practice can be preschool and primary school institutions, where parents are typically involved in activities, thus also engaging adults. Rituals engaging human children and sheep create long-term memories and mutual bondings that eventually sustain the commoning of the NBS.

### 4.3. Eco-compensation

Eco-compensation is currently a popular regulatory approach to avoiding or minimizing harm done to the environment by human economic activities ( Gastineau *et al.*, 2021; Wang *et al.*, 2022). Formally, this is a case of reciprocity: Humans claim a part of nature and must give something back. As a formal principle, this includes a wide range of actions, which do not necessarily imply that a functional equivalent of the original state of nature is restored, depending on how reciprocity is formally defined. There are scenarios where the link between harm and compensation is remote, such as clearing a forest in Europe and buying offsets for forestation in the Global South. Most thinking on eco-compensation is economical in that some notion of equal value is invoked, though not necessarily in monetary terms: a salient example is carbon offsets, where the quantity of carbon is the standard of value.

The perspective on ritual changes the approach to eco-compensation radically and directly reframes it in terms of NBG of NBS. Let us discuss this by taking urban land as an example, thus continuing with our discussion of property ( Hiedanpää *et al.*, 2023). The land is a resource that is part and parcel of the local ecosystem, and even if there is no legal recognition, all members of that ecosystem enjoy possession of the land. If urban green space is redeveloped, for example, into a parking lot, humans harm all other members at that location by confiscating their possessions. The redevelopment could be treated along the lines of legal prescriptions on public requisitioning of land, now including the interests of non-human stakeholders ( Hernandez-Santin *et al.*, 2023). However, for example, financial compensation is only applicable to humans.

**Figure 3.**
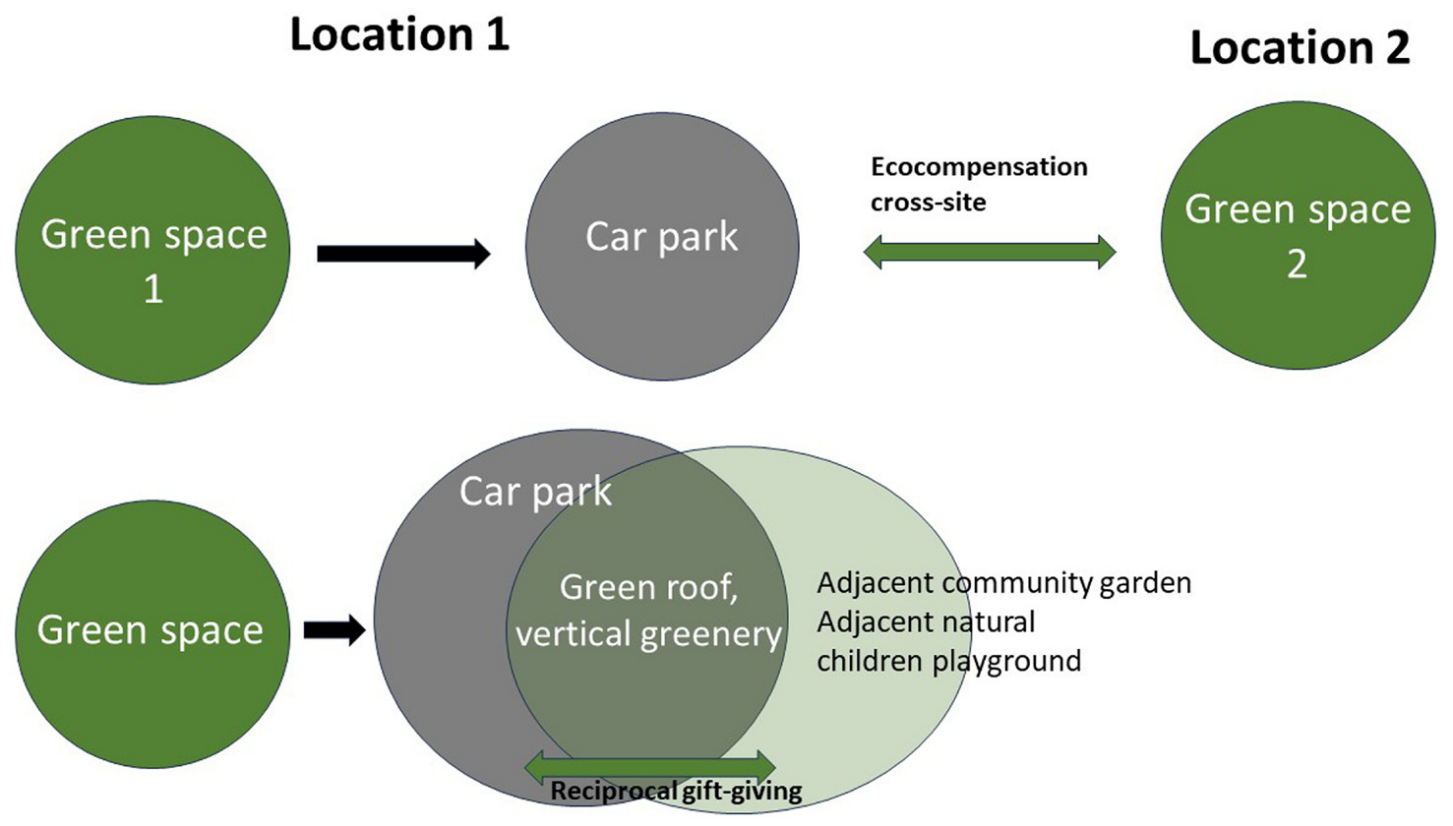
Eco-compensation: Institutional versus ritual governance.

In the ritual approach of NBG, we conceptualize the human appropriation of green space as the reciprocal relationship of gift exchange. The ritual notion of gift exchange is that gifts never lose their attachment to the original gift-giver, in the sense that they remain imbued by her identity and that the gift serves the purpose of expressing and cementing an enduring relationship between the gift-giver and -taker ( Hénaff, 2002). The critical difference to the regulatory notion of eco-compensation is that claiming green space for the human-built environment does not give something back to the ecosystem of equal value. That compensation even severs the link between humans and the ecosystem. In building a parking lot, the identity of the local ecosystem permanently changes. Hence, there is no way to compensate for this harm, for example, by creating a green space in another part of the city (see Figure 3, top). A genuine reciprocal exchange must be enacted in the same ecosystem, matching with ritual obligations of mutual gifts flow. Interestingly, this argument can be supported by considering the role of humans who are also suffering from the intervention and cannot enjoy benefits from eco-compensation, for example, young children and older adults who previously enjoyed the green space and cannot get access to the compensatory area at another place in the city. For them, destroying the green space means a permanent change in their way of life.

The ritual approach requires enacting local reciprocity ( Figure 3, bottom). In the example of the parking lot, one solution is to employ a biophilic architectural design of the parking lot ( Evans & Hardman, 2023). One option is to build a multi-story car park with vertical greenery, green roofs, and adjacent greenery that forms an alternative integrated green space that would match the needs of ecosystem members in the original green space. In general, this example points to the possibility of implementing a reciprocal exchange in which harm done to the local ecosystem is reciprocally balanced by designing the 'grey' artifacts to offer support and nurturing for nature. In a more detailed design, this would manifest in various ritual forms, such as neighbourhood stewardship for plants and animals living in the microhabitat or seasonal neighbourhood festivals. This might especially engage the vulnerable groups with no alternatives in the conventional eco-compensation approach. The example shows how ritual and reciprocity can even encompass the literally ‘hard’ case of greening grey infrastructure. There are many examples of how citizens engage even in the restoration of industrial sites or contaminated areas ( Ricci, 2022): Boyer (2024) speaks of ‘infrastructural citizenship’.

## 5. Discussion and conclusion

The concept of NBG rests on two pillars. The first is the peculiar anthropology of embodiment, that is, a distinct view of human nature that eschews disembodied approaches to governance that emphasize rational and abstract design and generalized incentives. This corresponds to the empirical record that governance works best when it is locally contextualized and inclusive in terms of local practices and personal engagement of people, as seminally elaborated by Elinor Ostrom ( Ostrom, 2015), and many others. As we have argued, this can be systematically grounded in a wider range of theoretical resources which have not yet been arranged together systematically. The second pillar of NBG is to tear down the walls of speciesism in thinking about institutions and to develop forms of rule-based multi-species interaction in which non-humans enjoy recognition, voice and representation ( Meijer, 2019). We have claimed that ritual is the paradigmatic form.

In the discourses about the roles of indigeneity in environmental governance, one approach is to recognize and reinstate Indigenous rituals and meanings in current practices ( Bush *et al.*, 2023). However, this would unduly constrain the view on ritual. The first step is to acknowledge the ubiquity of ritual across all societies, including Western late modernity: Just think of the rituals devoted to celebrities or sports events, political rituals, or the rituals of consumption, such as the public releases of the latest digital device. This acknowledgment triggers the switch of the Necker cube from disembodied institutions to embodied rituals as a generic and universal form of governance.

The question is how we can employ ritual in the practice of environmental governance, as we sketched in our NBS examples. Ritual is often associated with religious and quasi-religious stances, which we cannot imagine becoming a ‘policy tool.’ As said, one way is to tie up with existing traditions, which can offer rich inspiration, such as mentioned, Japanese Shinto. Another example is Chinese Fengshui that can inspire landscape design ( Yu, 2019). We generalize over these examples in referring to ‘place shaping’ as a distinct process that can nurture ritualization of reciprocity between people and nature, since eventually all ecological relationships are local in nature ( Grenni *et al.*, 2020). Landscape is a distinct notion that merges human subjectivities with ecological materialities ( Waldheim, 2016), and many human activities in landscapes (such as just walking) offer the potential for ritualization, such as inspiring concerns about nature and the eventual readiness to assume active roles, such as stewardships.

In principle, ritual can be seen as an object of design, such as deliberately creating a secular ritual for a specific purpose (the welcoming of newborn sheep is an example) ( Gordon-Lennox, 2017). However, there is no easy road to convincing people to follow a newly promoted ritual in terms of habitualization and embodiment. One powerful approach to ritualisation is creating ritual affordances by means of artful design of the environment in which actions unfold: An example is creating urban forests as objects of art, such as the ‘Aula verde’ ( Conte *et al.*, 2024) or supporting the rewilding of cities by creating nesting places for wild birds which are aesthetically attractive for both species ( Parker *et al.*, 2022). This stands in line with the concept of sensory governance. In fact, the instrumentalization of aesthetics is standard lore in modern business and marketing. Often, the problem is that the everyday aesthetics that emerge as a societal practice even block ecologically sensible practices (such as tastes for greenery that impoverish biodiversity) ( Saito, 2007). Hence, rethinking the material design of the human environment is strongly complementary to activating ritual as NBG: NBG downplays rational governance but highlights aesthetic, that is, sensory governance.

Art is well recognized as a tool in promoting eco-social transformation, mainly as a means to create awareness, to mobilize and to inspire ( Pearson *et al.,* 2018). In NBG, artful design becomes the key means to govern behaviour ( Herrmann-Pillath *et al.*, 2023). Art creates ritual affordances that can have dual functions, in the sense of semiotic co-option ( Maran, 2020), that is, multiple forms of interpretation in humans and non-humans, such that spontaneous coordination of interactions and eventually ritualization emerges. A simple and well-known example is to adapt features of buildings to the needs of birds, such as adding birdhouses, or paying attention to architectural features that allow for resting, bathing and so on. Once the presence of birds is widely recognized, a potential for ritualization emerges, such as welcoming migratory birds returning to the place. In other words, semiotic co-option means that a certain material assemblage arranges affordances for human and non-human actions that converge co-creating the place as natureculture of cohabitation ( Raymond *et al.*, 2017; Raymond *et al.*, 2018).

Therefore, a promising approach to activating ritual for NBG is artful design of the material environment, including the human-built, and less the invention of new ritual practices. This is similar to the behavioural economics approach to nudging and recognizes forms of distributed agency unfolding in ritual practices. Artful design creates ritual affordances, and which specific practices emerge, is left to more-than-human creative responses.

## Ethical approval and consent

Ethical approval and consent were not required.

## Data Availability

No data are associated with this article.
