# Supplementary material for: Rituals as Nature-Based Governance of reciprocity between people and nature
Source: Open Res Eur. 2024 Aug 7;4:66. Originally published 2024 Apr 12. [Version 2] doi: 10.12688/openreseurope.17206.2 (PMC11399756; doi:10.12688/openreseurope.17206.2)
Supplement: Peer review history

# Reviewer report: Reviewer response for version 2

**DOI:** 10.21956/openreseurope.19742.r42877

**Published:** 2024-09-13

**Authors:** Ronald Trosper

**Affiliations:** 1 The University of Arizona, Tucson, Arizona, USA

The revision addresses the concerns I raised about the first version.  This version clarifies the role of ritual in governance in useful ways.  Urban applications are important to demonstrate the relevance of the argument.

Is the argument persuasive and supported by appropriate evidence?

Partly

Is the work clearly and cogently presented?

Yes

Is the topic of the essay discussed accurately in the context of the current literature?

Partly

Does the essay contribute to the cultural, historical, social understanding of the field?

Yes

Reviewer Expertise:

Indigenous peoples and relationality, with a special focus on world views and economics.

I confirm that I have read this submission and believe that I have an appropriate level of expertise to confirm that it is of an acceptable scientific standard.

# Reviewer report: Reviewer response for version 2

**DOI:** 10.21956/openreseurope.19742.r42876

**Published:** 2024-08-28

**Authors:** Cecily Maller, David Kelly, Rebecca Olive

**Affiliations:** 1 Centre for Urban Research, RMIT University, Melbourne, Melbourne, Australia; 2 RMIT University, Melbourne, Victoria, Australia; 3 Centre for Urban Research, School of Global, Urban and Social Studies, RMIT University, Australia, RMIT University, Melbourne, Victoria, Australia

Thank you for the opportunity to review a revised version of this article. The clarity of the author's positionality and the structure of the paper are improved from the initial version and we found the arguments to be more convincing.

- The focus on densely populated cities in the Global South enables a clearer contribution to be made as does the emphasis on finding practical solutions to the bifurcation of people from nature in these (and other) contexts.
- We still have some reservations about a tendency to homogenize and generalize in some of the claims and arguments made (e.g., 'something is fundamentally wrong in our relationship with nature', depends on who the 'our' is referring to in this context. This would not be true of all people in all places). However, we accept that the solutions focus and the desire for upscaling inherently demands some form of generalization.
- Some points are still opaque or assume knowledge on the part of the reader (e.g., 'the discourse on indigeneity'). Overall, the paper is improved enough to warrant acceptance and the idea of nature-based governance holds merit.
- We look forward to future work that will interrogate and build on these ideas further. 

Is the argument persuasive and supported by appropriate evidence?

Partly

Is the work clearly and cogently presented?

No

Is the topic of the essay discussed accurately in the context of the current literature?

Partly

Does the essay contribute to the cultural, historical, social understanding of the field?

Partly

Reviewer Expertise:

NA

We confirm that we have read this submission and believe that we have an appropriate level of expertise to confirm that it is of an acceptable scientific standard.

# Reviewer report: Reviewer response for version 1

**DOI:** 10.21956/openreseurope.18595.r40100

**Published:** 2024-05-31

**Authors:** Cecily Maller, Rebecca Olive, David Kelly

**Affiliations:** 1 RMIT University, Melbourne, Victoria, Australia; 2 Centre for Urban Research, School of Global, Urban and Social Studies, RMIT University, Australia, RMIT University, Melbourne, Victoria, Australia; 3 Centre for Urban Research, RMIT University, Melbourne, Melbourne, Australia

Since their first appearance, robust discussion about the merits and limitations of ‘nature-based solutions’ has developed in a range of disciplines concerned with the sustainability, equity, and climate adaptability of cities. The value of these discussions is in their ability to constructively critique NBS and inform their design and implementation, as well as to posit potentially alternative pathways for making cities more habitable for humans and other species.

 We agree, as would many others, with the author’s claim that environmental protection policies and practices have failed to protect biodiversity and halt habitat loss. This article speaks to these and other issues by positing a potentially novel reframing of NBS with a mechanism of nature-based governance (NBG) premised on the concept of ritual, as one key form among other possibilities suggested. If we have understood correctly, NBG is defined as a means with which to ‘embed more-than-human practices’ in NBS. The idea that NBS would benefit from overarching principles or mechanisms of governance has merit because, if generally agreed to by governments, policymakers and program designers, it could help ensure that the design and implementation of NBS stay true(r) to their ideal claims of benefiting both people and nature (as is often stated).

 However, despite finding the aims and some claims of the paper tantalising, the way they are currently constructed is likely to struggle to convince readers, and overall, the paper risks missing an opportunity to make a clear contribution to knowledge.

 Below we offer some suggestions aimed at strengthening the conceptual clarity and structure of the paper that we hope the author finds useful in revising the article. As reviewers, we draw on our combined, complementary expertise across more-than-human critiques of NBS and the policy implications of relational theories (Maller), how everyday, recreational sport and leisure activities are experienced through cultural politics, ecological encounters, relationships with humans and non-humans (Olive), and how state and policy ecologies affect the capacity of humans and non-humans to dwell well (Kelly).

 At the outset, it would be useful if the target audience (who the paper is aimed at) could be made clearer to explain or highlight where, how and why the author is aiming to make a contribution. As part of this, we suggest that a few sentences be dedicated to explaining the author’s positionality regarding ontology and epistemology. By this we mean that it could make clearer to readers which research paradigms their work is aligned with, and their stance regarding what counts as knowledge. This sharing of positions is highly relevant to any scholarship that claims to be more-than-human and transdisciplinary.

 There are multiple concepts in the paper that are drawn on to construct an argument that ritual in NBG will reform the institutions that oversee NBS. The institutionalised context is implied but not actually discussed in full. However, more importantly, the six strands of literature discussed to expand/test the definition of NBG are given only a cursory introduction and it is not apparent what the problems are with each in order to support the claim at the bottom of page 6 that there is overwhelming evidence that the ‘standard view of institutions needs rethinking.’

 The literatures chosen to be discussed here are presented without any insight into the decision making behind their selection and this section, therefore, could be read as being comprised of concepts that have been ‘cherrypicked’. A more detailed explanation of how they fit together or contribute to the definition of NBG proposed would be helpful here. The lack of disciplinary situation of the work leads to further confusion as many of these terms would be defined differently in different literatures. Hence the need for positionality and clarity of the target audience mentioned above.

 Overall, the conceptual framing of NBG would benefit from minimising or streamlining the number of concepts in use while deepening the discussion, explanation and situation of the concept of ritual as a form of NBG in relevant literature. The concept of ‘ritual’ can remain central but we suggest deepening and expanding upon its conceptualisation and how it can be used in the context of NBG for NBS. Although there are limits to what can be covered in a single paper, the discussion of ritual occurs with only limited inclusion of other possible relevant scholarship within which it could be situated.

 We are not familiar with traditions of anthropological and sociological literature on ‘ritual’, but we would draw the author’s attention to examples including how does ritual differ (or compare) to social practices (e.g., Shove et al., 2012; Schatzki, 2002), habits (Hobson, 2003; Southerton, 2012), or other forms of reciprocity (e.g., as discussed in Indigenous and First Nations scholarship such as Bawaka Country et al., 2015; Kimmerer, 2013; Tynan, 2021). Further, we suggest the article needs more detailed situation in literature that entails discussions of human/non-human relationality, including examples from outside of Europe, and to explain how these systems would translate as NBG in Western governance frameworks and institutions. A specific example of this is how the ‘flow of gifts idea’ does not account for potential harms and conflicts that can arise in multispecies relations, nor on the importance of reciprocity. We draw the author's attention to Robin Wall Kimmerer’s influential (2013) book, Braiding Sweetgrass, and Sophie Chao’s (2022) book, In the Shadow of Palms, on this topic.

 There are several claims in the paper that are not well substantiated. For example, the statement that the paper presents ‘a radical and daring claim’ by arguing that ritual is a more-than-human practice performed by people and other species ignores extensive literature in disciplines that have focused on multispecies relations and/or posthumanist understandings of the world and embodiment, such as science and technology studies (e.g., Braidotti, 2021; Haraway, 2016; Tsing, 2015), environmental philosophers (e.g., van Dooren, 2022), human and cultural geography (e.g., Walker , 2021), planning (e.g., Houston et al. 2018; Steele, 2022), policy (e.g., Lea, 2020), as well as extensive ecofeminist writing (e.g., Plumwood, 2002; Rose, 2011). Furthermore, in the sections with the examples, these also do not support the claims to ‘radicalness’, particularly the example given of the carpark. Redesigning carparks to include plants does not discourage the reliance on cars as a key form of transport.

 A revised structure would also help strengthen the argument and give it more clarity. For example, the discussion on ‘ritual’ is fragmented throughout the various sections but would benefit from one comprehensive definition and discussion to establish the authors use of this concept for readers. Regarding other structural aspects of the paper, we recommend expanding the theoretical discussion and minimising or removing the examples which, as indicated above, currently do not serve the main arguments well.

 In terms of property and legal frameworks, the most compelling claim of the discussion was the section on Indigenous land claims in colonised countries. We had hoped for more connection with alternative models for how people connect with places and notions of connection, such as those hinted at in the mention of Shinto practices, but which were not developed. Expanding these ideas would be helpful in making a stronger argument in relation to animal and more-than-human “property” rights. We also recommend looking into literature on this topic from Aotearoa New Zealand, as well as literature challenging the notion of language being the sole domain of humans that is emerging in ecological research about critters including cetaceans and corvids.

 We agree that deeper commitment to reciprocity in NBS is an area in need of development and that, if done well, could move NBS beyond criticisms that they are transactional and human centric. However, at this stage the proposal that ritual is a way to bring reciprocity to the fore in NBS or NBG is unconvincing and lacking evidence.

 In summary, we recommend: 1) clarifying the audience of the paper and the inclusion of a brief description of the author’s positionality; 2) streamlining the number of concepts in use while deepening the discussion of those concepts, particularly, explanation and situation of the concept of ritual in relevant literature; 3) toning down the claims made (or number of claims) and 4) rethinking the use/inclusion of the examples as part of a major restructure.

 We hope these suggestions and recommendations are useful in clarifying and better justifying the arguments and assisting the author in making a more decisive contribution.

Is the argument persuasive and supported by appropriate evidence?

Partly

Is the work clearly and cogently presented?

No

Is the topic of the essay discussed accurately in the context of the current literature?

Partly

Does the essay contribute to the cultural, historical, social understanding of the field?

Partly

Reviewer Expertise:

NA

We confirm that we have read this submission and believe that we have an appropriate level of expertise to state that we do not consider it to be of an acceptable scientific standard, for reasons outlined above.

## References

[1] : Working with and learning from Country: decentring human author-ity. *cultural geographies* .2015;22(2) : 10.1177/1474474014539248 269-283 10.1177/1474474014539248

[2] Reference source

[3] In the Shadow of the Palms/authors.

[4] Staying with the Trouble/authors.

[5] : Thinking Habits into Action: the role of knowledge and process in questioning household consumption practices. *Local Environment* .2003;8(1) : 10.1080/135498303200041359 95-112 10.1080/135498303200041359

[6] : Make kin, not cities! Multispecies entanglements and ‘becoming-world’ in planning theory. *Planning Theory* .2018;17(2) : 10.1177/1473095216688042 190-212 10.1177/1473095216688042

[7] Braiding Sweetgrass INDIGENOUS WISDOM, SCIENTIFIC KNOWLEDGE AND THE TEACHINGS OF PLANTS/authors.

[8] : Wild Policy.2020; 10.1515/9781503612679 10.1515/9781503612679

[9] : Feminism and the Mastery of Nature.2002; 10.4324/9780203006757 10.4324/9780203006757

[10] The Site of the Social/authors.

[11] : Shove, Pantzar and Watson. The dynamics of social practice: everyday life and how it changes. *Nordic Journal of Science and Technology Studies* .2016;1(1) : 10.5324/njsts.v1i1.2125 41-43 10.5324/njsts.v1i1.2125

[12] : Habits, routines and temporalities of consumption: From individual behaviours to the reproduction of everyday practices. *Time & Society* .2013;22(3) : 10.1177/0961463X12464228 335-355 10.1177/0961463X12464228

[13] : Wild infrastructure. *Current Opinion in Environmental Sustainability* .2022;55: 10.1016/j.cosust.2022.101171 10.1016/j.cosust.2022.101171

[14] The Mushroom at the End of the World: On the Possibility of Life in Capitalist Ruins/authors.

[15] : What is relationality? Indigenous knowledges, practices and responsibilities with kin. *cultural geographies* .2021;28(4) : 10.1177/14744740211029287 597-610 10.1177/14744740211029287

[16] A World in a Shell Snail Stories for a Time of Extinctions/authors.

[17] Energy and Rhythm Rhythmanalysis for a Low Carbon Futur/author.

# Response

**Published:** 2024-07-12

**Authors:** Carsten Herrmann-Pillath

**Affiliations:** Max Weber Centre for Advanced Cutural and Social S, University of Erfurt, Erfurt, Thuringia, Germany

I followed the reviewers’ advice to restructure the paper substantially. I summarize this in my general report about the revision. With this thorough revision, I aim to clarify the focus on governance as the topic of the paper. This is important as the reviewers are skeptical about my use of the term “radical” and refer to many other strands of the literature that may appear as even more “radical.” I toned down a bit, but I maintain my claim: My reference is the established literature on governance, and here, introducing ritual as a new governance mode is indeed radical. As I stated in the beginning (the reviewers invited me to do so), I am writing as an economist and concerned about how to introduce, sustain and upscale NBS in cities. The places I have in mind are not townships or suburban areas close to rural areas but ‘hard cases’ such as the Chinese megacity Shenzhen, where I am also doing fieldwork. This is why I keep the car park example: These are not affluent North American or Australian (sub)urban settlements with a population partly open-minded to ideas of greening their lifestyles. Grey infrastructure is dominant and is often seen as the pinnacle of progress and prosperity. Building the case for NBS while telling people to give up their cars (which may even go electric yet need a car park) is a political non-starter. My paper combines radicalism in rethinking governance with gradualism in ecosocial transformation, facing the backlash against green policies everywhere, such as in my country, Germany, which is sadly associated with extreme right populism. A new pivot in my paper is that I discarded the paragraphs on sketching the various theoretical inspirations, thus engaging with the respective comments by the reviewers. Instead of that, I have thoroughly restructured section 2. First, there is a separate subsection in the definition of ritual, which is extended, and second, I introduce a new theoretical frame that distinguishes between embodied and disembodied reciprocity, thus establishing embodiment as the key theoretical concept. About the previous, section 2.2 on governance now includes a discussion of infrastructural governance, which has recently seen matching theoretical innovations (such as sensory governance). I hope these substantial changes meet the reviewers’ expectations about deepening the discussion of key concepts. The distinction between embodiment and disembodiment is what stays at the theoretical centre now and builds the bridge between references to indigeneity and the settings that I mentioned above. indigeneity is invoked by some of the key references that reviewers 2 point out, such as Kimmerer, mentioned by both. Embodying reciprocity is the generic form beyond specific cultural frames, which avoids the frictions and difficulties in transferring culturally contextualized frames to other societies: To put it with a critical tone, Kimmerer is a privileged tribal intellectual, a professor of botany, and lives close to nature in a country richly endowed with spatial resources that allow for many forms of nature contact. For me, the key message of her book is that it is not the specific form of indigenous spirituality that creates relationality, but the enactment of rituals that are nature-inclusive, such as rituals that conceptualise the relationship between people and nature as mutual gift-giving. The challenge is, I repeat, how to transfer this idea to contexts like an extremely densely populated urban village in Shenzhen where people struggle in the capitalist treadmill from dusk to dawn. Most practices described in the Kimmerer book are not accessible to these people, while they are certainly a group most vulnerable to climate change and in need of NBS. Given these concerns, I decided to put more detail into the stylized NBS cases and locate them in the literature, such as on stakeholder approaches. I condensed the property section into a few paragraphs in the new theoretical section on reciprocity to illustrate the difference between embodied and disembodied forms of governance which are salient in the role of mapping territory, a topic much emphasized by Sophie Chao.

# Reviewer report: Reviewer response for version 1

**DOI:** 10.21956/openreseurope.18595.r40092

**Published:** 2024-05-30

**Authors:** Ronald Trosper

**Affiliations:** 1 The University of Arizona, Tucson, Arizona, USA

The article proposes that ritual be given a core role in nature-based governance (NBG). Since both humans and nonhumans use ritual as they interact with each other, the proposal has merit as a way to break down the human/nature divide that causes so much trouble in relationships with nature. Ritual occurs in many Indigenous societies as part of their implementation of reciprocity between humans and nature.  Such reciprocity would thus have a core role through implementation of rituals.

 I have answered “partly” regarding the use of current literature and presentation of evidence.  The paper omits the connection between rituals and the formation of relationships.  That distinction is also questioned in the literature on relationality.  Donati and Archer (2015) expand the idea of “actor” from individuals to “relational subjects”. A group of persons form a relational subject when they have one or more common goals and when they pay attention to the needs and ideas of each other through the practice of reflexivity. Reflexivity is the ability to consider one’s own goals in the context of other entities and their goals.  It operates through internal and external conversations.  When a group of people create a relational subject, they do so by sharing in relational goods such as trust, equity, friendship, solidarity, and identity.  Ritual plays a key role in creating and maintaining such relational goods.  The paper comes close to utilizing the idea of relational subject when it cites literature on “assemblages of individuals and material entities.”

 That relationality matters for a great many fields is also argued by Eyster, Satterfield and Chan (2023).  They credit relational thinking to Indigenous cosmologies (p. 459). They argue that relationships are fundamental; “relationships create the world” (p. 464).

 Each of the author’s examples can be interpreted in a relational frame.  Property literature has long emphasized that property is a relationship with a thing in the context of relations among humans.  Standard property theory assumes things are inanimate; if they are animate, then relationships must take their viewpoints and actions into account. Ritual does that.  Proposals for ecosystem compensation work to maintain environmental functions when specific local relations are emphasized, rather than long-distance and disconnected compensation schemes.  Similarly, wildfire is a property of relationships among humans, animals, and plants.  The paper uses the specific role of grazing animals in controlling fuel loads; the analysis could be extended to other uses of relationships to control fuel loads and make fire useful rather than destructive.  The paper also recognizes the role of gardening in changing people’s perceptions and relationship with nature, a point also developed by Kimmerer (2013).

 A reason ritual is so important is that it is a key part of making and maintaining relationships.  Ritual is a way for humans to communicate with nonhumans and include them in relational subjects.  As Donati and Archer emphasize, relational thinking is completely different from the Individualist/holism dichotomy that pervades much of social science. The relational order of reality is not situated between individualism and holism; it is a completely different way to view the world (p. 32).  This is consistent with the author when he states “In ritual, the analytical distinction between the actors and the institution becomes obsolete.”  (p. 5)

 The concluding section of the paper proposes that artful design is a way to activate ritual in nature-based governance.  A better approach is to build relational subjects that rely on ritual in their formation and in their interaction.  The result is that nature-based governance is relational governance.

Is the argument persuasive and supported by appropriate evidence?

Partly

Is the work clearly and cogently presented?

Yes

Is the topic of the essay discussed accurately in the context of the current literature?

Partly

Does the essay contribute to the cultural, historical, social understanding of the field?

Yes

Reviewer Expertise:

Indigenous peoples and relationality, with a special focus on world views and economics.

I confirm that I have read this submission and believe that I have an appropriate level of expertise to confirm that it is of an acceptable scientific standard, however I have significant reservations, as outlined above.

## References

[1] : The Relational Subject.2015; 10.1017/CBO9781316226780 10.1017/CBO9781316226780

[2] : Empirical examples demonstrate how relational thinking might enrich science and practice. *People and Nature* .2023;5(2) : 10.1002/pan3.10453 455-469 10.1002/pan3.10453

[3] Reference source

# Response

**Published:** 2024-07-12

**Authors:** Carsten Herrmann-Pillath

**Affiliations:** Max Weber Centre for Advanced Cutural and Social S, University of Erfurt, Erfurt, Thuringia, Germany

The emphasis on relationality is important and motivated my new theoretical distinction between embodied and disembodied reciprocity which is a major extension of the paper. The new argument approaches ritual as a type of action that enacts embodied reciprocity grounded in relationality as a subjective cognitive and emotional state. However, I did not refer to relational self theory because I have myself worked extensively on these topics elsewhere (Basso and Herrmann-Pillath 2024 and earlier work) where I refer to the distinction between we-mode and I-mode made by Tuomela. These are complex theoretical issues that I cannot possibly tackle in a single paper. I do not start out from relationality as foundational term (instead using embodiment) because there is the danger overextending its reach, what I see in the Eyster paper, which produces an expansive list of approaches with “flavours of relationality”. For example, social network theory in sociology is clearly relational, but is completely lacking any reference to specific emotional and cognitive states or values. Hence, I regard relationality as a state (my we-mode) that can be realised in specific contexts. One means of producing such contexts is ritual governance. I understand that Professor Trosper agrees with that. Accordingly, I do not use the term ‘relational governance’. Relational governance seems a more general concept that might, for example, apply only for human-human relationships. NBG is specific to relationships between humans and nature aka other species. I hope that I could make this point clearer in the many edits of phrases and even new paragraphs: NBG is about including nature in the human domain of governance as a ‘governing’ force. NBG is also now standard term in the COEVOLVERS project of which this paper is a part, so I cannot change this expression. My treatment of art is motivated by the concern that we cannot just invent rituals and invite people to join. We must create aesthetic affordances that elicit creativity in inventing rituals on the grassroots level.
